# Indole-3-Acetic Acid Is Produced by *Emiliania huxleyi* Coccolith-Bearing Cells and Triggers a Physiological Response in Bald Cells

**DOI:** 10.3389/fmicb.2016.00828

**Published:** 2016-06-08

**Authors:** Leen Labeeuw, Joleen Khey, Anna R. Bramucci, Harjot Atwal, A. Paulina de la Mata, James Harynuk, Rebecca J. Case

**Affiliations:** ^1^Department of Biological Sciences, University of AlbertaEdmonton, AB, Canada; ^2^Department of Chemistry, University of AlbertaEdmonton, AB, Canada

**Keywords:** algae, auxin, cell type, coccolith, *Emiliania huxleyi*, haptophyte, indole-3-acetic acid (IAA), phytohormone

## Abstract

Indole-3-acetic acid (IAA) is an auxin produced by terrestrial plants which influences development through a variety of cellular mechanisms, such as altering cell orientation, organ development, fertility, and cell elongation. IAA is also produced by bacterial pathogens and symbionts of plants and algae, allowing them to manipulate growth and development of their host. They do so by either producing excess exogenous IAA or hijacking the IAA biosynthesis pathway of their host. The endogenous production of IAA by algae remains contentious. Using *Emiliania huxleyi*, a globally abundant marine haptophyte, we investigated the presence and potential role of IAA in algae. Homologs of genes involved in several tryptophan-dependent IAA biosynthesis pathways were identified in *E. huxleyi*. This suggests that this haptophyte can synthesize IAA using various precursors derived from tryptophan. Addition of L-tryptophan to *E. huxleyi* stimulated IAA production, which could be detected using Salkowski's reagent and GC × GC-TOFMS in the C cell type (coccolith bearing), but not in the N cell type (bald). Various concentrations of IAA were exogenously added to these two cell types to identify a physiological response in *E. huxleyi*. The N cell type, which did not produce IAA, was more sensitive to it, showing an increased variation in cell size, membrane permeability, and a corresponding increase in the photosynthetic potential quantum yield of Photosystem II (PSII). A roseobacter (bacteria commonly associated with *E. huxleyi*) *Ruegeria* sp. R11, previously shown to produce IAA, was co-cultured with *E. huxleyi* C and N cells. IAA could not be detected from these co-cultures, and even when stimulated by addition of L-tryptophan, they produced less IAA than axenic C type culture similarly induced. This suggests that IAA plays a novel role signaling between different *E. huxleyi* cell types, rather than between a bacteria and its algal host.

## Introduction

Small signaling molecules are important components of inter-species interactions, and have been shown to play a role in multicellularity, settlement, and pathogenesis (Joint et al., 2002; Manefield et al., 2002; Matsuo et al., 2005; Schaefer et al., 2008). While these interactions occur at the cell-to-cell interface, they can have large-scale effects at the community and ecosystem levels (Charlson et al., 1987; Vardi et al., 2009; Cooper and Smith, 2015). Many of these small molecules are specific in their mode of action, and can be restricted to a specific sub-population of cells in an organism. Current research indicates that signaling molecules are important components of inter-kingdom communication in bacterial-host systems (Hughes and Sperandio, 2008). One such group of small molecules, which is well-studied in plants, is the phytohormone indole-3-acetic acid (IAA). IAA is one of the most abundant and important plant auxins, and also among the first plant hormones to be discovered (Went, 1926; Teale et al., 2006). Auxins are responsible for growth and development, such as in tissue differentiation, fertility, cell division, orientation, and enlargement in terrestrial plants (Lau et al., 2009; Zhao, 2010; Finet and Jaillais, 2012). Although auxins were originally thought to exist only in plants, their biosynthetic pathway has since been characterized in bacteria and fungi (Spaepen et al., 2007). However, much of what is known about IAA's physiological roles comes from studies of plants' responses to exogenous IAA (Sakata et al., 2010). Indeed, IAA has commercial use and is frequently sprayed over agricultural fields to increase crop yields (Sudha et al., 2012).

At elevated levels, IAA can cause physiological damage. For instance, IAA stimulates the production of ethylene, which inhibits plant growth (Xie et al., 1996). It has also been implicated in pathogenesis of plants, as bacterial symbionts have the potential to take over the plant biosynthetic pathway (Yamada et al., 1985; Yamada, 1993) and produce it in plant hosts (Patten and Glick, 2002; Gravel et al., 2007). The diverse effects on the host (ranging from stimulation to pathogenesis) depends on the amount of free IAA produced, as well as the host's sensitivity to this compound (Spaepen et al., 2007). Pathogenic bacteria can alter host metabolic processes for their own benefit, such as by up-regulating the production of IAA, which produces uncontrolled cellular division leading to gall disease in plants (Escobar and Dandekar, 2003). A well-studied example of this pathogenic strategy is the crown gall disease caused by *Agrobacterium tumefaciens*, which involves the formation of galls on the lower stem and roots of infected plants (Zhu et al., 2000; Escobar and Dandekar, 2003). *A. tumefaciens* hijacks the infected plant cells to promote unregulated growth, resulting in the formation of galls, which act as nutrient factories producing amino acids and sugar derivatives that the pathogen uses for energy (Zhu et al., 2000).

In algae, bacterially-produced auxins have been implicated in bud induction in the macroalgal rhodophyte *Gracilaria dura* (Singh et al., 2011). Roseobacters, a clade of marine α-proteobacteria, have been shown to produce IAA or alter its production by a host. Bacterial cells identified as roseobacters using FISH have been localized to the intercellular spaces within galls, in the red macroalga *Prionitis lanceolata* (Ashen and Goff, 2000). These galls have elevated levels of IAA; however it is not known if the bacterium or algal host produce the IAA as neither has been isolated as an axenic culture (Ashen et al., 1999). Both micro- and macroalgal chlorophytes have shown sensitivity and increased growth when exposed to exogenous IAA (Jin et al., 2008; Salama et al., 2014). Amin et al. (2015) recently demonstrated the role IAA plays between the unicellular diatom *Pseudo-nitzschia multiseries* and its associated bacterial community, specifically looking at the roseobacter, *Sulfitobacter* sp. SA11. They showed an up-regulation of tryptophan synthesis in the alga led to a corresponding increase in IAA production by the bacterium, which in turn acted as an inter-kingdom signaling molecule that increased growth of *Pseudo-nitzschia* (Amin et al., 2015). It's perhaps serendipitous that roseobacters were first coined marine *Agrobacterium* as the similarity of the roseobacter—algae interaction to the *A. tumefaciens*—plant host system is striking (Ahrens and Rheinheimer, 1967). Another roseobacter, *Ruegeria* sp. R11, is a known algal pathogen and produces IAA (Case et al., 2011; Fernandes et al., 2011; Mayers et al., 2016), although the role IAA plays in this interaction (if any) is unknown.

The question of whether eukaryotic algae produce IAA remains largely unresolved (Cooke et al., 2002; Lau et al., 2009; Ross and Reid, 2010). Commercially, addition of algal extracts has been claimed to induce growth responses in plants characteristic of auxins (Crouch and Van Staden, 1993). A vast body of studies spanning from the 1940s to the present have reported IAA in various unicellular and multicellular forms of brown, green, and red algae (Van Overbeek, 1940; Jacobs et al., 1985; Sanderson et al., 1987; Tarakhovskaya et al., 2007; Lau et al., 2009). The different lifestyles and forms of multicellular macroalgae compared to unicellular microalgae suggest that IAA would play a different signaling role in these organisms, as cell growth, fertility, and development occur within a macroorganism and between microorganisms within a population. The differences of IAA signaling at the intra-and inter-organismal level will be key to understanding its role in micro- and macroalgae.

In macroalgae, IAA was detected in the brown algae *Fucus distichus* and *Ectocarpus siliculosus* (Basu et al., 2002; Le Bail et al., 2010), as well as in the red algae *Pyropia yezoensis* and *Bangia fuscopurpurea* (Mikami et al., 2015). *Nitella*, which is part of Charophyta, the algal phylum most closely related to land plants has also been suggested to produce IAA. This suggests that primitive auxin metabolism was present at least as early as the ancestor of land plants and charophytes (Sztein et al., 2000). De Smet et al. (2011) found putative auxin biosynthesis and auxin transporter orthologs encoded in unicellular chlorophyte genomes. IAA has also been detected in various microalgal chlorophytes, including *Chlorella pyrenoidosa* and *Scenedesmus* spp. (Mazur et al., 2001; Prieto et al., 2011). These studies have found evidence corroborating the hypothesis that evolution of auxin biosynthesis predates the divergence of land plants and some algal taxa. However, many of these studies used non-axenic algal cultures, and as some algae-associated bacteria produce auxins (Evans and Trewavas, 1991; Fernandes et al., 2011; Bagwell et al., 2014; Dittami et al., 2014; Amin et al., 2015), it remains unknown if these studies are reporting auxin concentrations from the alga itself or its bacterial epiphytes. Furthermore, the concentration of IAA observed in some studies may not be high enough to play a role in algal development (Lau et al., 2009). It is possible that multi-step IAA extractions underestimates the auxin concentration due to degradation of IAA during the procedure (Mazur et al., 2001). Auxins could also be concentrated within specific algal structures or cells, making whole plant extractions an underestimation of local concentrations within the alga. Nonetheless, Lau et al. (2009) and Ross and Reid (2010) expressed reservations regarding evidence for IAA biosynthesis by algae described in literature, especially microalgae, which they reported to lack auxin signaling pathways homologous to those present in land plants.

Using bioinformatics and empirical experimentation, this study aims to elucidate the presence and function of IAA in a unicellular algal species, *Emiliania huxleyi*. This microalga is a small (5 μm), globally abundant haptophyte, part of a different eukaryotic supergroup than all other algae so far investigated for the presence of IAA (Archibald, 2009). It is also a major primary producer in oceans and plays a substantial role in the carbon and sulfur cycles (Holligan et al., 1993). It has three distinct cell types: the non-motile diploid bald cells (type N), the coccolith producing cells (type C), and the haploid motile type cells (type S) (Klaveness and Paasche, 1971; Laguna et al., 2001).

We identified homologs for the genes of several complete tryptophan dependant IAA biosynthesis pathways in the genome of *E. huxleyi*. To confirm this genotype, axenic cultures of C and N type *E. huxleyi* cell types were screened for the production of IAA after stimulation with L-tryptophan. These cell types were also exposed to exogenous IAA to look at their phenotypic response. Interestingly, only C type cells were able to produce IAA and only N type cells had a phenotypic response to it. To verify if the known IAA producer, the roseobacter *Ruegeria* sp. R11, could also influence its host through this signaling molecule, the two organisms were co-cultured. R11 and *E. huxleyi* grown together produce less IAA than *E. huxleyi* grown alone (with stimulation by tryptophan addition in both cases). The lack of response to the bacterium able to produce IAA, combined with the differential production and response to this signal according to cell type, is unlike what has been previously observed in any other algae.

## Materials and methods

### Genomic survey of tryptophan dependant IAA biosynthesis pathways

Sequences for enzymes in the tryptophan dependant IAA biosynthesis pathways of *Arabidopsis thaliana*, which were used to query algal databases for homologs, were obtained from Le Bail et al. (2010). Query sequences to search for the bacterial IAA biosynthesis genes were obtained from Spaepen et al. (2007). BLASTp searches for algal homologs were performed and up to three representative completed genomes were searched for each major algal group (chlorophytes, rhodophytes, glaucophyte, diatoms, pelagophyte, brown algae, eustigmatophyte, chrysophyte, dinoflagellate, haptophyte, and cryptophyte) (Table 1). Additionally, the *E. huxleyi* genome was specifically surveyed for homologs of plant signaling and transport proteins, using sequences from Le Bail et al. (2010) as queries. Available complete roseobacter genomes (as listed on http://www.roseobase.org) and the genome of *Ruegeria* sp. R11 (from which IAA production has been identified) (Fernandes et al., 2011) were surveyed. For all homolog searches, a bi-directional best hit (BBH) BLASTp search of the resulting hits was performed against the organism(s) from which the query sequenced was obtained. An *e*-value of 10^−10^ or less for the BLASTp and the BBH BLASTp search was used as a cut-off for homology (Le Bail et al., 2010; Kiseleva et al., 2012; Mikami et al., 2015).

**Table 1:** **Distribution of IAA biosynthesis genes in algal genomes**.

	**YUCCA**	**AMI1**	**TAA1**	**CYP79B2**	**CYP79B3**	**AAO1**	**CYP71A13**	**TDC**	**MYR1**	**SUR1**	**SUR2**	**NIT1**
**LAND PLANTS**												
*Arabidopsis thaliana*	AEE86075	Q9FR37	Q9S7N2	NP_195705	NP_179820	Q7G193	O49342	Q8RY79	P37702	O65782	Q9SIV0	AEE77887
**GREEN ALGAE**												
*Ostreococcus sp RCC809*^a^	−	−∕+	−	−∕+	−∕+	−	−	−	−∕+	−∕+	+	−
*Coccomyxa subellipsoidea*^a^	−	+	−	−∕+	−∕+	+	−	+	−∕+	−∕+	−∕+	+
*Chlamydomonas reinhardtii*^a^	−	+	−	−∕+	−∕+	+	−	+	−∕+	−∕+	−	−
**RED ALGAE**												
*Cyanidioschyzon merolae*^b^	−	−∕+	−	−	−	−	−	−	−	−	+	−
*Porphyridium purpureum*^c^	−	−	−	−	−	−	−	−	−	−	−	−
*Chondrus crispus*^d^	−	−∕+	−	−∕+	−∕+	+	−	−	−∕+	−∕+	+	−
**GLAUCOPHYTES**												
*Cyanophora paradoxa*^d^	−	−	−	−	−	−	−	−	−	−	−	−
**DIATOMS**												
*Fragilariopsis cylindrus*^a^	−	−	−	−	−	−	−	−	−	−	−	−
*Phaeodactylum tricornutum*^a^	−	−∕+	−	−∕+	−∕+	+	−	−	−∕+	−∕+	+	−∕+
*Pseudo-nitzschia multiseries CLN-47*^a^	+/−	−∕+	−	−	−∕+	+	−	−	−∕+	−∕+	+	−
**PELAGOPHYTE**												
*Aureococcus anophagefferens*^a^	−	−∕+	−	−∕+	−∕+	+	−	−	−	−	+	−
**BROWN ALGAE**												
*Ectocarpus siliculosus*^d^	−	+	−	+	+	+	−	+	+	+	+	−
**EUSTIGMATOPHYTES**												
*Nannochloropsis oculata*^d^	−	−	−	−	−	−	−	−	−	−	−	−
**CHRYSOPHYTES**												
*Ochromonas danica*^d^	−	−	−	−	−	−	−	−	−	−	−	−
**DINOFLAGELLATES**												
*Symbiodinium minutum*^e^	−	−	−	−	−	−	−	−	−	−	−	−
**CRYPTOPHYTES**												
*Guillardia theta*^a^	+/−	−∕+	−∕+	−∕+	−∕+	+	−	−	−	−∕+	−∕+	−
*Hemiselmis andersenii*^d^	−	−	−	−	−	−	−	−	−	−	−	−
***HAPTOPHYTES***												
*E. huxleyi CCMP1516*^d^	+/-	−∕+	−∕+	−∕+	−∕+	+	−	+	−∕+	−∕+	+	+

*Presence is determined initially by a bi-directional best hit (BBH) BLASTP hit with an E-value < 1 × 10^−10^ or less using the characterized land plant A. thaliana gene as a query, as well as correct functional prediction using ESG and orthology with the plant enzyme as determined by OrthoMCL. (+) indicates that it matches all three criteria, (−∕+) indicates it is confirmed by ESG but not OrthoMCL, while (+∕−) indicates confirmation by OrthoMCL but not ESG. The following databases were searched: JGI (^a^), http://merolae.biol.s.u-tokyo.ac.jp/blast/blast.html (^b^), Bhattacharya et al. (2013) (^c^), NCBI (^d^), and OIST Marine Genomics Unit (http://marinegenomics.oist.jp/genomes/gallery/) (^e^). The abbreviations used for the enzyme can be described as follows: YUCCA, Tryptamine monooxygenase; AMI1, Indole-3-acetamide hydrolase; TAA1, Tryptophan amino transferase; CYP79B2 and CYP79B3, Cytochrome P450s; AAO1, Indole-3-acetaldehyde oxidase; CYP71A13, Indole-acetaldoxime dehydratase; TDC, Tryptophan decarboxylase; MYR1, Myrosinase; SUR1, C-S lyase; SUR2, CYP83B1; and NIT1, Nitralase*.

The sequences were assigned to orthologous groups using OrthoMCL (Li et al., 2003), where the hits grouping with the query sequence were considered positive. Functional prediction using the ESG package (Chitale et al., 2009) was concurrently performed and only homologs which had confidence scores similar to or higher than the predicted function of the query sequences, were considered positive.

### Algae and bacteria strains

Axenic *Emiliania huxleyi* strains CCMP2090 (bald N cell type), and CCMP3266 (coccolith producing C cell type) were obtained from the Provasoli-Guillard National Centre for Marine Algae and Microbiota (NCMA). The *E. huxleyi* strains were maintained in L1-Si medium made using natural filtered seawater (Bamfield Marine Science Centre, BC, Canada) (Guillard and Hargraves, 1993) at 18°C in a diurnal incubator (12:12 h dark-light cycle). The algal cultures and L1-Si medium were checked for bacterial contamination by microscopy and by inoculation onto ½ marine agar (18.7 g Difco Marine Broth 2216 supplemented 9 g NaCl and 15 g Difco agar in 1 L) followed by incubation at 30°C for 2 d. However, this screening can't exclude the possibility of contaminating unculturable bacteria that are not readily visualized with microscopy. The algae were grown statically for 5 d to 10^4^ cells/mL (early-log) for experiments.

The bacterium *Ruegeria* sp. R11 was maintained at 30°C on ½ marine agar plates then grown on a rotating drum to stationary phase in 5 mL ½ marine broth (18.7 g Difco Marine Broth 2216 supplemented 9 g NaCl in 1 L) for 24 h before experiments.

### Algal growth experiments with tryptophan, IAA, and *Ruegeria* sp. R11

L- and D-tryptophan (Sigma-Aldrich, St. Louis, MO, USA) were freshly prepared for each experiment in L1-Si medium and filter sterilized. *E. huxleyi* was grown in 0.02 mM NaOH, 1 and 2% ethanol solutions in L1-Si medium as these were the final concentrations in experiments where they are used as the solvent for IAA. The 1% ethanol solution had no observable effect on algal cells while the 2% ethanol and 0.02 mM NaOH resulted in a decreased chlorophyll concentration and potential quantum yield. Subsequently, IAA was prepared in 50% ethanol-water solution and all IAA additions were made to a final 1% ethanol concentration in L1-Si medium. The compound screening and co-cultivation experiments were performed as previously described (Bramucci et al., 2015). Briefly, *E. huxleyi* strains (CCMP2090 and CCMP3266) were supplemented with 1, 0.1, and 0.01 mM L-tryptophan and 0.1 mM D-tryptophan in L1-Si medium. IAA was added to CCMP2090 and CCMP3266 at a concentration of 0.1, 0.01, and 0.001 mM in L1-Si medium and controls of 0 and 1% ethanol. There was no significant difference between the 0 and 1% ethanol controls, so data analysis uses the 1% ethanol as the control as treated samples include 1% ethanol (from the IAA stock solution). Wells were randomly assigned in 48-well plates (Becton, Dickinson and Company, Franklin Lakes, NJ, USA) with triplicate samples for each condition such that each plate contained all the samples for a single time point. *E. huxleyi* had an initial concentration of 10^4^ cells/mL for all treatments.

For the bacterial-algal co-cultures, bacterial cells were washed twice by centrifugation and re-suspended in L1-Si medium, before being diluted to the initial concentration of 10^4^ CFU/mL. Co-cultures were performed with and without 0.1 mM L-tryptophan. All microtiter plates were incubated in a diurnal incubator (12:12 h dark-light cycle) at 18°C for all experiments.

### Microscopy

Algal cells were visualized throughout the experiment using an Axio Imager M2 microscope (Zeiss, Oberkochen, Germany) and processed using Zen 2012 (Zeiss).

### Fluorescence measurements

To measure the chlorophyll fluorescence and photosynthetic yield, samples were taken at the mid-point of their dark cycle (5–7 h into the dark cycle) and diluted in L1-Si medium to within the detection range using a pulse-amplitude-modulation (PAM) fluorometer (WATER-PAM, Waltz, Effeltrich, Germany). A dark adaption period of 3 min was determined, after which a saturating pulse was applied and the fluorescence readings were taken to calculate the minimal dark fluorescence (F_0_) that is directly correlated to the chlorophyll content, the maximum dark fluorescence (F_m_) and the Photosystem II (PSII) potential quantum yield (F_v_/F_m_) (F_v_/F_m_ = (F_m_ –F_0_)/F_m_) (Schreiber et al., 1986; Kooten et al., 1990). Three-microtiter wells were sampled (and not re-sampled) as replicates at each time point to determine the yield. Data were processed using SigmaPlot 12. Statistical significance was determined using a one-way ANOVA and Student-Newman-Keuls (SNK) test.

### Biomass and IAA measurements

Samples were taken from sampled well at each time point and biomass measured (OD at 680 nm) using a Synergy H1 microplate reader (BioTek, Winooski, VT, USA). A colorimetric test was used to determine if IAA, indole-3-pyruvic acid, or indole-3-acetamide is produced as previously described (Glickmann and Dessaux, 1995). In brief, samples were taken and centrifuged (10 min at 5000 rpm for algae and 2 min at 14,000 rpm for bacteria) then the supernatant was mixed in a 1:1 volume ratio with fresh Salkowski reagent [12 g FeCl_3_ (EMB Millipore, Billerica, MA, USA) in 1 L of 7.9 M H_2_S0_4_ (Sigma-Aldrich)]. Samples were then incubated in the dark for 30 min at room temperature. The OD (530 nm) and emission spectra for peak wavelength were measured. The IAA concentration of L1-Si medium was measured using the Salkowski reagent and sterile molecular water as the blank to determine if the seawater in L1-Si medium was a potential source of IAA; L1-Si medium did not contain a detectable IAA concentration. Subsequently L1-Si medium was used to blank all readings. IAA concentrations were prepared in L1-Si medium to construct a standard curve to determine the IAA concentration from samples. Statistical significance was determined using a one-way ANOVA and SNK test.

### Flow cytometry

Algal samples were fixed with 0.15% glutaraldehyde (Sigma-Aldrich) for flow cytometry by incubating cells in the dark for 10 min, then flash-frozen in liquid nitrogen and stored at −80°C until flow cytometry was performed using a FACSCalibur (Becton, Dickinson and Company). A 488 nm laser was used for excitation. Samples were first run using chlorophyll fluorescence (670 nm) for detection. Membrane integrity was evaluated using Celltox Green (Promega, Madison, WI, USA) (520 nm). Data were processed using FlowJo 9.2.

### GC × GC-TOFMS

Replicate 100 mL cultures of CCMP2090 and CCMP3266 were grown in 250 mL Erlenmeyer flasks. Flasks were inoculated with 10^4^ cells/mL *E. huxleyi* in 0.1 mM L- or D-tryptophan and no tryptophan addition for the control. Cells were harvested at 16 d by centrifuging at 5000 rpm for 10 min.

IAA standards were created by adding 2.8 mM (~500 ppm) IAA to control algal samples. All samples were then extracted using an equal volume of methanol, ultrasonicated, and vacuum filtered on 1.6 μm Whatman 1820-047 GF/A, 47 mm diameter filters (Sigma-Aldrich) three times. This was then passed through a column of sodium sulfate (Sigma-Aldrich). Derivatization was done according to previous literature (Birkemeyer et al., 2003), whereby 5 μL of the extracted algal sample was added to 100 μL of *N*-methyl-*N*-(trimethylsilyl) trifluoroacetamide (MSTFA) (Sigma-Aldrich), then incubated at 90°C for 30 min. To determine if L1-Si medium contained IAA at a concentration below detection of the Salkowski reagent, 10 mL of L1-Si medium was tested. Additional treatment was needed as this extraction was performed on liquid (L1-Si medium) rather than biomass. First, the pH of the sample was lowered to ~1.5 using hydrochloric acid (Sigma-Aldrich), then the liquid sample was extracted by adding 1 mL methylene chloride (Sigma-Aldrich), vortexing vigorously, and removing the methylene chloride layer. This was repeated three times. The methylene chloride was then evaporated and 10 mL methanol was added. The algal extraction procedure was then followed following biomass concentration (i.e., filtration). To confirm the extraction worked, a sample of L1-Si medium was first spiked with IAA at ~6 mM, and the expected IAA peak was detected.

The samples were analyzed on a Leco Pegasus 4D GC × GC-TOFMS (Leco Instruments, St. Joseph, MI, USA). The columns used for the first- and second- dimensions were a 30 m × 0.25 μm, 1 μm film thickness Rtx-5MS (Chromatographic Specialties, Brockville, ON, Canada) and a 1.6 m × 0.25 mm, 0.25 μm film thickness Rtx-200MS (Chromatographic Specialties) respectively. Helium (5.0 grade; Praxair, Edmonton, AB, Canada) was used as the carrier gas with flow controlled at 1.5 mL/min. The analytes were desorbed in the split/splitless injection port of the GC × GC-TOFMS using an inlet temperature set at 230°C, operating in splitless mode. The 47 min GC method began with an initial oven temperature of 70°C for 1 min, followed by a ramp of 6°C/min up to 320°C, and ending with a 5 min hold in the first oven. Relative to the primary oven, the secondary oven was programmed to have a constant offset of +5°C and the modulator a constant offset of +15°C. The modulation period was 2.0 s. The TOFMS had an acquisition rate of 100 Hz and acquired over a mass range of m/z 10–700. The detector voltage was −1350 V, the ion source temperature was 200°C, and the MS transfer line temperature was 250°C.

GC × GC-TOFMS data were processed using ChromaTOF® (v.4.43; Leco). For processing, the baseline offset was set at the middle of the noise (0.5), the minimum S/N for the base peak and the sub-peaks were set at 10, and the data were auto smoothed by the software. The first dimension peak width was set at 14 s while the second dimension peak width was set at 0.15 s. Identifications of the compounds were made based on library matches with the NISTMS 2008 Library mass spectral database and relative retention index. In this case, a minimum match factor of 75% of the library was required before a name was assigned to a compound.

## Results and discussion

### Presence of tryptophan dependent IAA biosynthesis pathways in algae and roseobacters

IAA biosynthesis consists of a complex set of tryptophan dependent and independent pathways that is not yet fully understood (Zhao, 2010; Tivendale et al., 2014). The tryptophan independent pathway remains genetically undefined (Normanly, 2010; Nonhebel, 2015). In contrast, the tryptophan dependent pathways have been well studied in *A. thaliana* (Sztein et al., 2000; Zhao, 2010), with many of the genes encoding for its enzymes functionally described. Biosynthesis of IAA by bacteria has also been studied, but members of this domain are thought to use pathways and enzymes different than those found in plants (Spaepen et al., 2007). To investigate the presence of tryptophan dependent pathways outside of plants, public databases were searched using *A. thaliana* and known bacterial IAA biosynthesis genes as queries against algal and roseobacter genomes. Homologs of several of these genes were found in a wide array of algae (Table 1) and roseobacters (Supplementary Table 1). However, it should be noted that the presence of homologs does not necessarily translate to the presence of the bona-fide functional enzyme involved in this pathway, especially for those that fall under a functionally broad protein family (such as CYP79B2 and CYP79B3 that are cytochrome P450s). To determine which homologs were likely to have a conserved function with plant enzymes, functional prediction was performed with the ESG package (Chitale et al., 2009) and homologs were categorized in orthologous groups using OrthoMCL (Li et al., 2003). Only homologs part of the same orthologous groups as the plant enzymes and/or with identical functions predicted with ESG were included (Table 1 and Supplementary Tables 1, 2). Patterns of co-occurring genes suggesting the presence of complete tryptophan dependent pathways were found in some algae, and genes encoding partial pathways in others (Table 1). In algae in which only partial pathways were found, IAA could potentially be produced as a result of the completion of essential biochemical steps by their proximal bacterial symbionts (Dittami et al., 2014). We also found homologs of some bacterial IAA biosynthesis genes in algae (Supplementary Table 2), suggesting that alternate pathways for IAA biosynthesis to those found in plants might be present.

**Figure 1:**
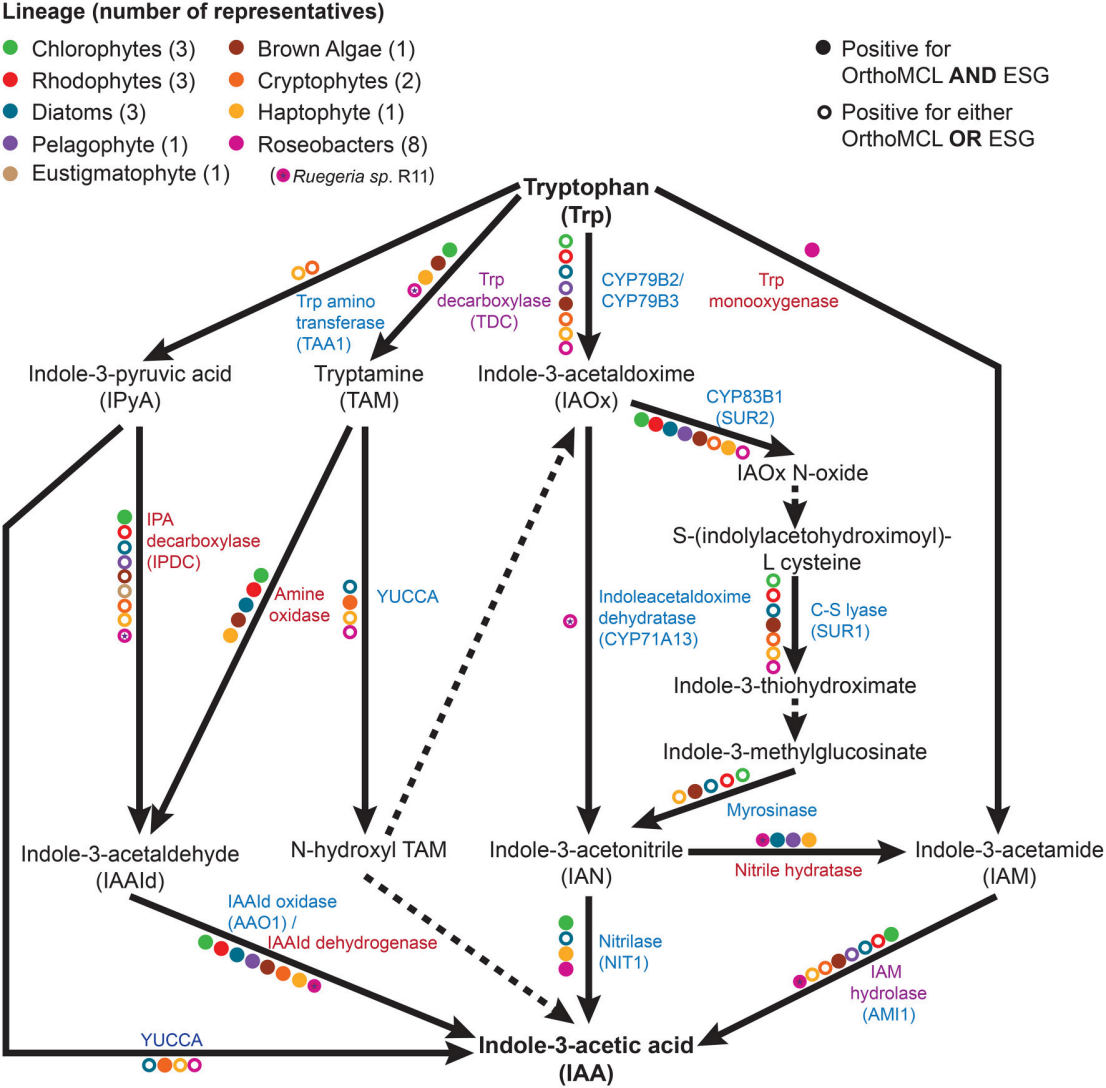
**The tryptophan dependent indole-3-acetic acid (IAA) biosynthesis pathways**. Metabolic reactions catalyzed by known enzymes are indicated with solid lines while dashed lines indicate a currently unknown enzyme. Listed in blue are the enzymes with a plant seed sequence, while in red are the enzymes with a bacterial seed sequence. Listed in purple are the enzymes that are found in plants, algae, and bacteria. Colored dots represent the presence of the given enzyme in at least one member of a specific taxonomic group, as indicated by bi-directional best hit (BBH) BLAST search (minimum *e*-value of 10^−10^) followed by Ortho MCL and ESG functional prediction.

As shown in previous studies (Kobayashi et al., 1993; Amin et al., 2015), the roseobacter genomes encode for the genes known in bacteria to be involved in the synthesis of IAA through the indole-3-acetamide (IAM) pathway (Figure 1). There is a possibility that roseobacters are capable of synthesizing IAA through another pathway, as they contain a putative tryptophan decarboxylase that produces the IAA precursor tryptamine, a putative indole-3-pyruvate decarboxylase, as well as indole-3-acetaldehyde (IAAd) dehydrogenase, which converts the tryptamine derivative IAAd to IAA (Supplementary Table 1). However, Amin et al. (2015) highlighted the difficulty in determining whether the IAAd dehydrogenase found in roseobacters is specific to IAA biosynthesis or used in other pathways. Nitrile hydratase and IAM hydrolase, which can together convert indole-3-acetonitrile (IAN) to IAA, have also been identified in roseobacters ((Fernandes et al., 2011); Figure 1 and Supplementary Table 1). However, there is currently no known pathway in bacteria that can produce the IAN this enzyme pair uses as an initial substrate. Interestingly, the genes to produce IAN from tryptophan (the indole-3-acetaldoximine, or IAOx, pathway) are widely distributed in algae (Figure 1). This complementarity could form the basis of a symbiosis between algae and roseobacters enabling the production of IAA. This symbiosis has been suggested as the basis for the presence of IAA in the brown algae *E. siliculosus*, as the bacterial symbiont *Candidatus* Phaeomarinobacter ectocarpi contains the genes necessary to complete the synthesis of IAA in complementarity with the genes found in the alga (Dittami et al., 2014).

The survey of public sequence databases also revealed that the principal tryptophan dependent IAA biosynthesis pathway in plants, the indole-3-pyruvic acid (IPyA) pathway, using tryptophan amino transferase (TAA) and YUCCA (Mashiguchi et al., 2011), is not widely distributed in algae, if at all. It has been previously noted in genomic surveys that some chlorophytes have the genetic potential for IAA biosynthesis, but that the tryptamine (TAM) and IAOx pathways are the most likely candidates for the synthesis of this compound (De Smet et al., 2011; Kiseleva et al., 2012). More distantly related algae (outside the supergroup Archaeplastida, which contains plants, chlorophytes, and rhodophytes) have not been thoroughly investigated, except for the brown alga *E. siliculosus*, in which the IAOx and TAM pathways had the most supporting evidence for being at least partially present (Le Bail et al., 2010), although it should be noted that combining known bacterial and plant genes, at least one complete putative pathway is present (Figure 1). Surprisingly, phylogenetically diverse microalgae such as diatoms, haptophytes, and cryptophytes, have a wide distribution of putative enzymes enabling the production of IAA from tryptophan (Figure 1 and Table 1). The haptophyte *E. huxleyi*, for example has a complete putative pathway to yield IAA from tryptophan, combining plant and bacterial enzymes (using tryptophan decarboxylase, bacterial amine oxidase, and indole-3-acetaldehyde oxidase; Figure 1 and Supplementary Table 2). However, some of these enzymes, such as indole-3-acetamide hydrolase, may not be specific to the IAA biosynthesis pathway. Therefore, the identification of homologs does not necessarily mean that algae can produce IAA from tryptophan. The fact that this haptophyte contains homologs of all the genes for a complete putative pathway, as well as homologs of some genes involved in nearly every other tryptophan dependent pathway, makes it a good candidate for further investigation.

Putative homologs of genes belonging to the IAOx pathway seem to be the most widely distributed in algae, which in plants is limited to the Brassicaceae family (Mano and Nemoto, 2012). When putative IAA biosynthesis genes are found in an alga, they are not always present in every member of the group to which it belongs. For example, Mikami et al. (2015) did not find any homologs for IAA biosynthesis genes in bangiophycean red algae (not included in this study), while this study found homologs in cyanidiophyceaen and florideaphycean red algae (Table 1). Such a patchy distribution of the genes that could be involved in IAA biosynthesis within each algal group and between algal groups can be interpreted in two main ways. It could suggest that the basic mechanisms for IAA biosynthesis were present in the ancestor of terrestrial plants and archaeplastid algae, with multiple loss events occurring, or alternatively that the pathway was distributed by lateral gene transfer (LGT). These hypotheses have been greatly debated for the IPyA pathway, which is composed of the tryptophan aminotransferase and flavin-containing monooxygenase (YUCCA) enzymes, converting tryptophan in IAA via indole-3-pyruvic acid. LGT from bacteria to an ancestor of land plants was argued to be the most parsimonious explanation for the origin of IPyA pathway (Yue et al., 2014; Turnaev et al., 2015). Another view is that the pathway evolved earlier in the ancestor of land plants and charophytes (Wang et al., 2014, 2016). Interestingly, IAA biosynthesis genes found in bacteria but not plants were also found widely in algae, giving additional evidence for LGT events contributing to its evolution in eukaryotes (Supplementary Tables 1, 2). Further research would be needed to determine where the different tryptophan dependent IAA biosynthesis pathways originated and what role bacteria played in their evolution.

Although many algae may possess one or more tryptophan dependent IAA biosynthesis pathways (or parts of it), there has been little to no evidence to suggest that they contain the necessary receptor proteins known to facilitate IAA signaling and the polar auxin transport that is crucial in determining cell directionality in plants (Køeèek et al., 2009; Lau et al., 2009; De Smet et al., 2011). There have been recent studies suggesting a few possible homologs in the transcriptome and genome of brown and red algae (Le Bail et al., 2010; Wang et al., 2015). A survey of *E. huxleyi* did not identify any homologs of known response proteins, including: ARF (auxin response factors); PIN proteins (transmembrane proteins that actively regulate the transport and efflux of auxins); or the Aux/IAA transcriptional repressors. Only one hypothetical protein homologous to TIR1/AFB (transport inhibitor response/auxin signaling f-box) was found (XP_005761773, with an *e*-value of 3e^−18^). However, if IAA serves as a growth-promoting hormone in algal systems, it has been suggested that the AUX/IAA/ARF signaling pathway may not be required, and other signaling mechanisms may exist (Lau et al., 2009; Zhang and Van Duijn, 2014).

### *E. huxleyi* coccolith bearing (C) cells produce IAA when stimulated by tryptophan

**Figure 2:**
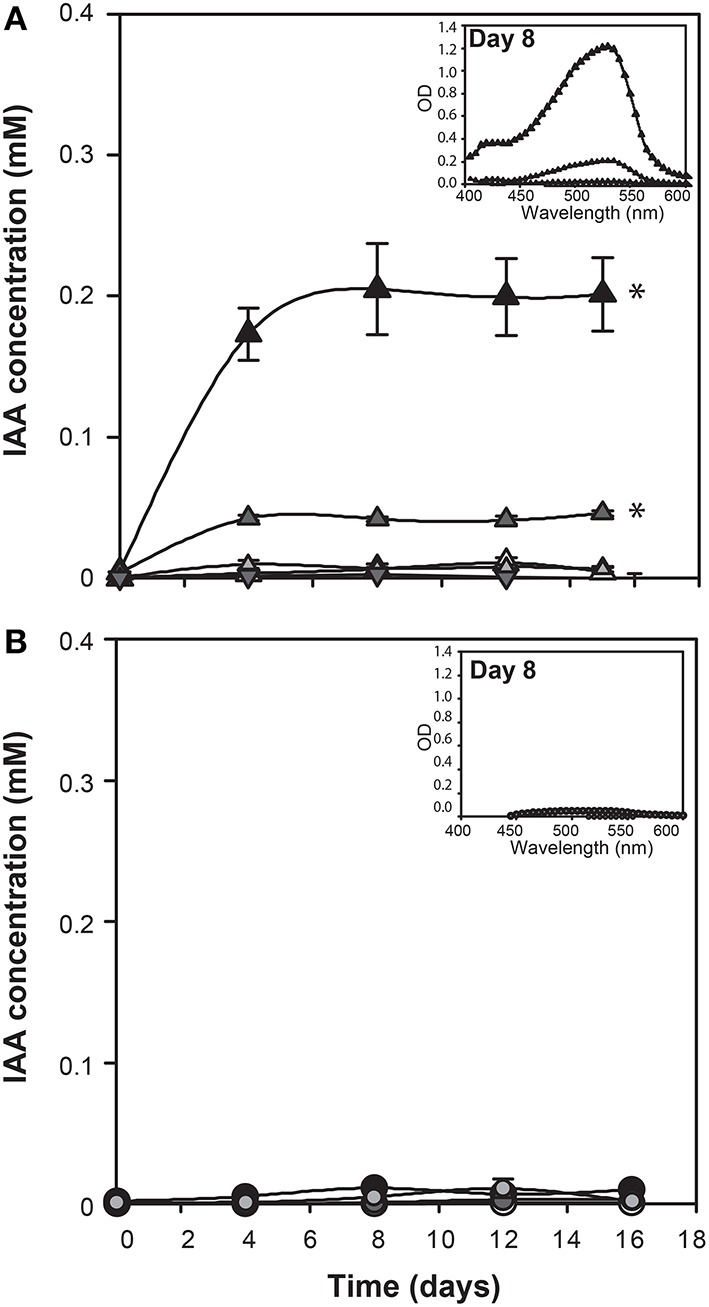
***E. huxleyi* production of indole-3-acetic acid (IAA) in the presence of various concentrations of tryptophan**. IAA concentration was derived from OD measurements of culture supernatants after addition of Salkowski reagent. The experiment was performed with coccolith bearing CCMP3266 **(A)** and bald CCMP2090 **(B)** *E. huxleyi* strains. Triangles represent CCMP3266 with various concentrations of L-tryptophan (black for 1 mM, dark gray for 0.1 mM, light gray for 0.01 mM, and white for the control with no L-tryptophan added). Inverted triangles represent CCMP3266 grown with 0.1 mM D-tryptophan. Using the same color scheme, circles represent CCMP2090 grown with various concentrations of L-tryptophan. Inset is the emission spectrum taken on 8 d to indicate the peak wavelength. Error bars represent ±1 SE. An asterisk (^*^) at a time point indicates that it is significantly different to the control and an asterisk at the end of the line indicates that the treatment is significantly different to the control.

Since genes encoding enzymes of tryptophan dependent IAA biosynthetic pathways are present in the *E. huxleyi* genome, L-tryptophan was added to axenic cultures of coccolith bearing C type (CCMP3266) and bald N type (CCMP2090) cells at various concentrations to determine if these strains would convert it to IAA. IAA was detected using the Salkowski reagent, a rapid colorimetric assay which recognizes indolic compounds, with IAA having an optimal peak of 530 nm (Glickmann and Dessaux, 1995). The lower limit of detection of the Salkowski reagent in this experimental set-up is 0.001 mM. While 1 mM L-tryptophan was inhibitory to the growth of CCMP3266 (Supplementary Figure 1), the Salkowski reagent indicated that the alga was converting the L-tryptophan into IAA at concentrations of L-tryptophan as low as 0.1 mM, with the characteristic color and optimal wavelength observed (Figure 2). IAA was detected soon after the addition of L-tryptophan, and its concentration stayed constant, reaching 0.1 mM when 1 mM of L-tryptophan was added, and 0.07 mM with 0.1 mM L-tryptophan. The addition of D-tryptophan, which should not stimulate IAA production (Baldi et al., 1991), yielded no detectable IAA (Figure 2).

Unlike CCMP3266, growth of CCMP2090 was not inhibited at 1 mM of L-tryptophan, although it did cause a slight decrease in PSII health (Supplementary Figure 1). However, when CCMP2090 was supplemented with tryptophan, IAA was not produced (Figure 2).

**Figure 3:**
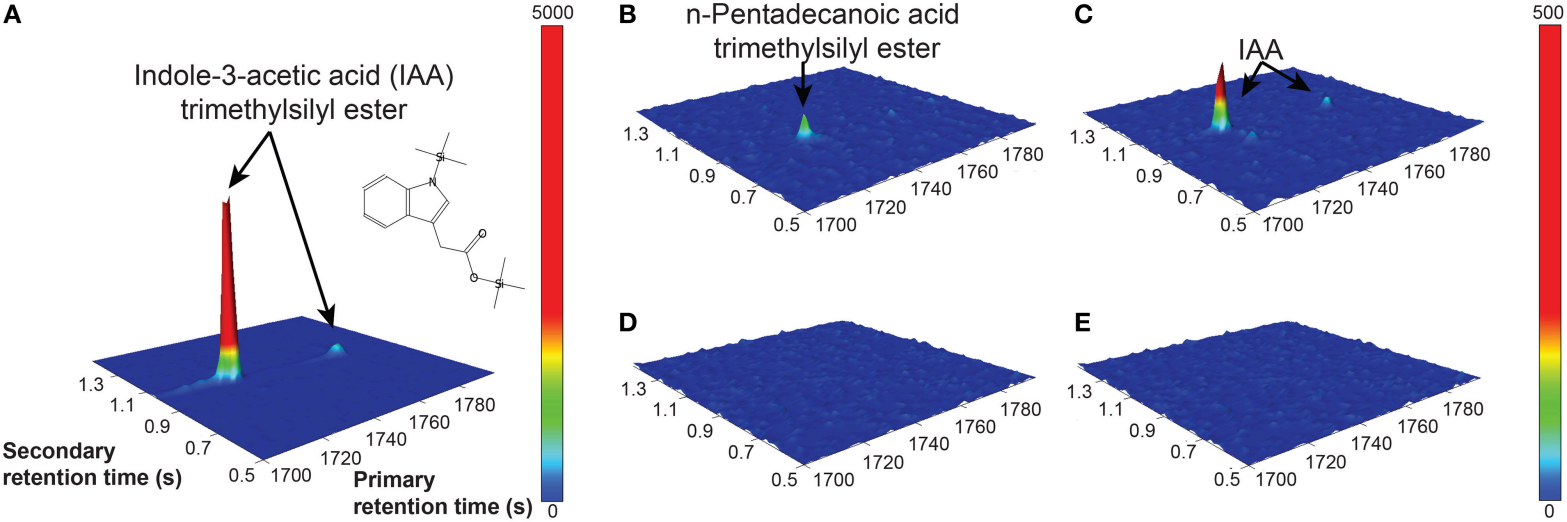
**GC × GC–TOFMS surface plots of selected mass channel (*m/z*) of 130**. A standard of 2.8 mM IAA is shown **(A)**, along with *E. huxleyi* C type culture (CCMP3266) control (no tryptophan added) **(B)**, CCMP3266 grown with 0.1 mM L-tryptophan **(C)**, *E. huxleyi* N type culture (CCMP2090) control (no tryptophan added) **(D)**, and CCMP2090 growth with 0.1 mM L-tryptophan **(E)**. Cells were harvested from cultures on 16 d. Identifiable peaks are labeled with the compound to which they correspond.

The Salkowski reagent is a rapid assay demonstrating the conversion of L-tryptophan to IAA, but use of the reagent in algal samples has been criticized due to its lack of specificity for IAA, as its absorption spectra can overlap with indole-3-pyruvic acid (Buggeln and Craigie, 1971; Glickmann and Dessaux, 1995). Consequently, we performed GC × GC-TOFMS analysis on the samples to determine whether the main auxin produced was indeed IAA, and/or to identify other auxins or intermediates if they were present. GC × GC-TOFMS was run on the harvested 16 d samples of CCMP3266 and CCMP2090 grown with 0.1 mM L- and D-tryptophan. Co-culture samples of *E. huxleyi* with R11 were not analyzed with GC × GC-TOFMS as the presence of R11 lowered the IAA concentration, as detected by the Salkowski reagent, when co-cultured with CCMP3266 compared to when it was grown alone. GC × GC-TOFMS is an analysis that facilitates detection of unknown compounds using the retention times in two dimensions and uses a library search for the resulting hits. It has several distinct advantages, including better separation of components, simplified sample preparation, increased peak capacity, and high selectivity and sensitivity (Dallüge et al., 2002; Adahchour et al., 2008). An IAA standard was added to algal samples and compared to the algal controls as well as samples with the addition of 0.1 mM of L- or D-tryptophan. Identification was based on the first and second retention time matching the standard, as well as the similarity (how well the peak matches the library) and reverse match (how well the library matches the peak) factors. The peak retention times and peak identification based on NISTMS 2008 Library mass spectral database determined the presence of IAA in the CCMP3266 sample containing L-tryptophan (Figure 3), and the absence of hits in the control, D-tryptophan supplemented sample, or any of the CCMP2090 samples (Table 2 and Figure 3). L1-Si medium did however contain a barely detectable peak with the same retention times as IAA (Table 2). However, peak area and the signal to noise ratio was very low (Table 2). Additionally, the volume of liquid medium used for the control extraction (10 mL) was much larger than that of the biomass subsample (0.1 mL), from which IAA was detected at a ~10,000 fold lower IAA signal per unit volume compared to CCMP3266 biomass. This provides strong evidence that the *E. huxleyi* C cells produces IAA when stimulated with L-tryptophan, but that N cells are unable to produce this compound when grown with or without L-tryptophan under the conditions tested.

**Table 2:** **GC × GC-TOFMS peak table**.

**Sample ID**	**Name**	R.T. (s) (1D^*^, 2D^**^)	**Peak area**	**Quant masses**	**Similarity**	**Reverse**	**Signal to noise (S/N) ratio**
IAA standard	3-Indoleacetic acid, trimethylsilyl ester	(1728, 1.020)	23810	73	816	846	812.56
2.8 mM	3-Indoleacetic acid, trimethylsilyl ester	(1774, 1.000)	218651	73	862	862	2760.90
CCMP3266	3-Indoleacetic acid, trimethylsilyl ester	(1728, 1.020)	1299	73	416	796	34.08
0.1 mM L-tryptophan	3-Indoleacetic acid, trimethylsilyl ester	(1772, 0.990)	17418	73	765	793	639.58
CCMP3266	No peak found						
0.1 mM D-tryptophan							
CCMP3266 Control	No peak found						
CCMP2090	No peak found						
0.1 mM L-tryptophan							
CCMP2090	No peak found						
0.1 mM D-tryptophan							
CCMP2090 Control	No peak found						
L1-Si Medium (seawater)	Unknown	(1772, 0.990)	290	73	NS^#^	NS	10.80

* *1D, 1^st^ Dimension*;

** *2D, 2^nd^ Dimension*;

# *NS, Not searchable*.

### Differential effect of exogenous IAA added to bald (N) and coccolith bearing (C) *E. huxleyi* cell types

**Figure 4:**
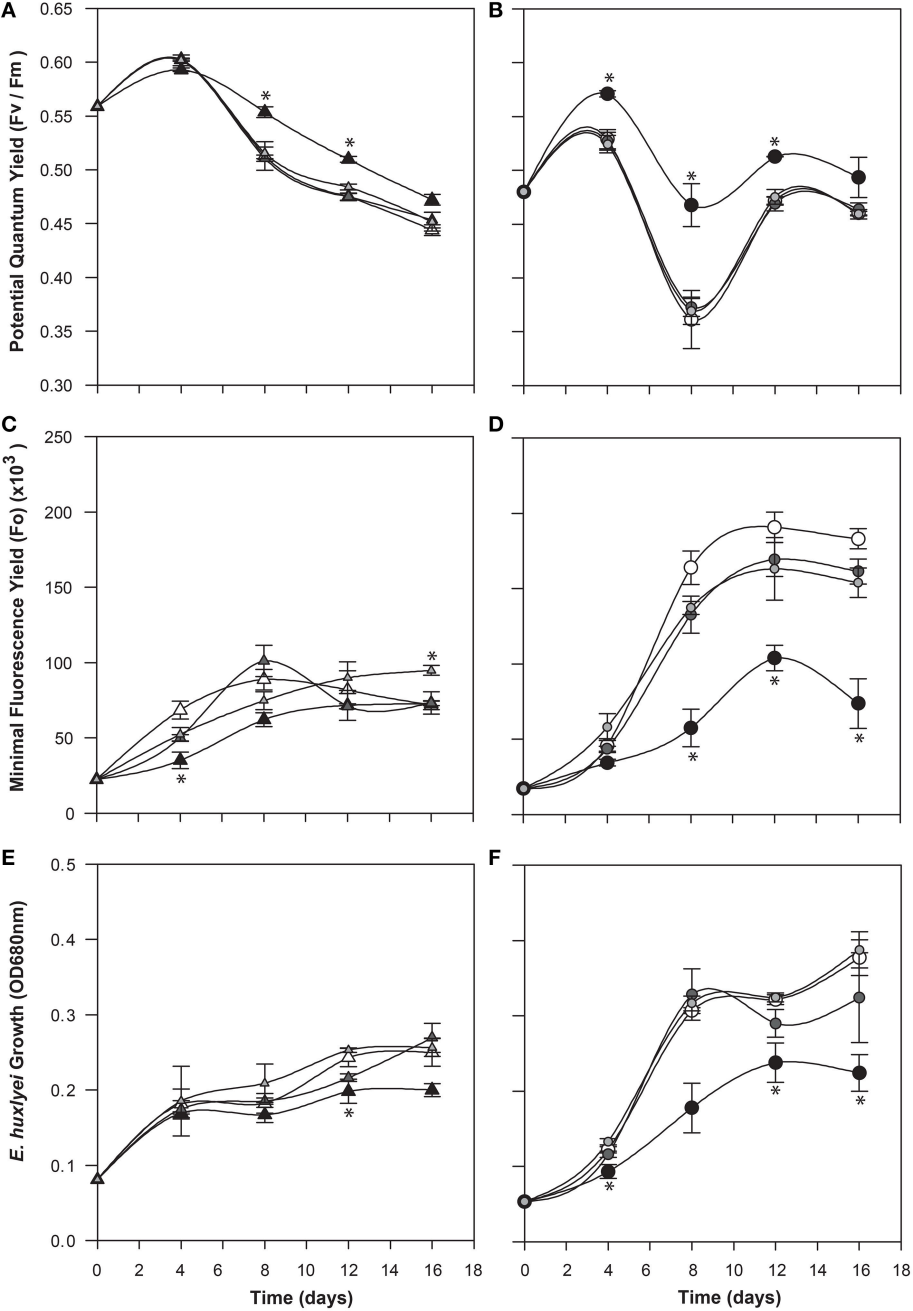
**Effect of exogenous indole-3-acetic acid (IAA) on the bald (CCMP2090) and coccolith bearing (CCMP3266) *E. huxleyi* strains**. The algae were co-cultured with concentrations of 0.1–0.001 mM of IAA [black for 0.1 mM, dark gray for 0.01 mM, and light gray for 0.001 mM, and white for the control (L1-Si medium with 1% ethanol)]. Triangles represent CCMP3266 while circles represent CCMP2090. The potential quantum yield of CCMP3266 **(A)** and CCMP2090 **(B)** with various concentrations of IAA is shown, as well as the minimal fluorescence for the two strains, CCMP3266 **(C)** and CCMP2090 **(D)**. Growth is displayed as the OD measurement at 680 nm for CCMP3266 **(E)** and CCMP2090 **(F)**. Error bars represent ±1 SE. An asterisk (^*^) at a time point indicates that it is significantly different to the control and an asterisk at the end of the line indicates that the treatment is significantly different to the control.

**Figure 5:**
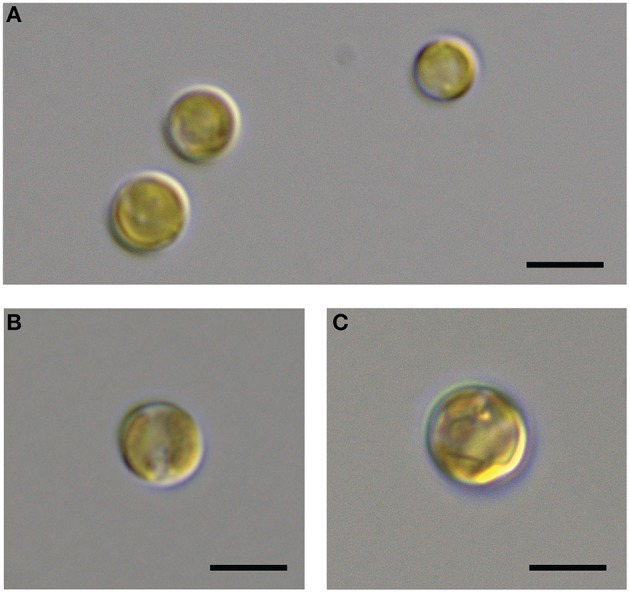
**DIC microscopic observation of the bald *E. huxleyi* strain (CCMP2090) exposed to indole-3-acetic acid (IAA)**. The CCMP2090 control is grown in L1-Si medium with 1% ethanol at 8 d **(A)**, the alga with 0.01 mM IAA at 8 d **(B)**, and the alga with 0.1 mM IAA at 12 d **(C)**. The scale bar represents 5 μm.

**Figure 6:**
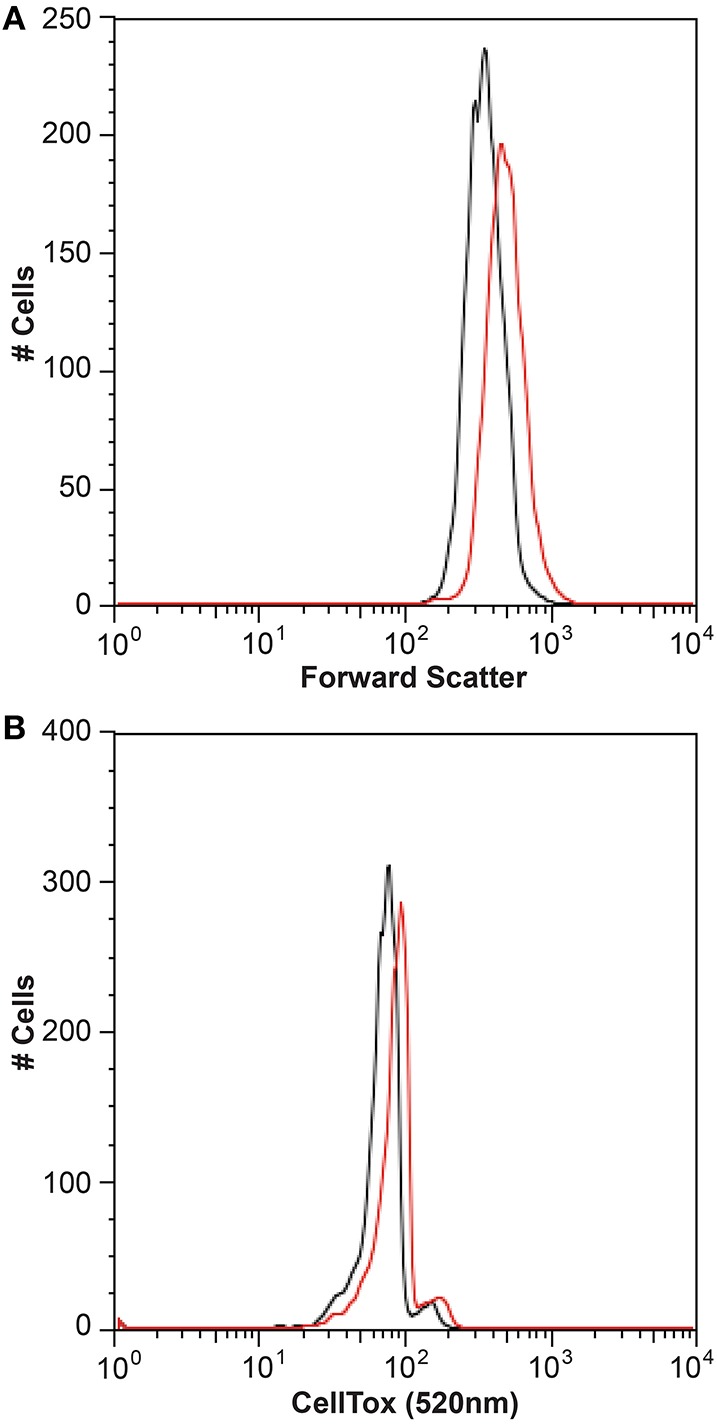
**Flow cytometry analysis of the bald *E. huxleyi* strain (CCMP2090) treated with indole-3-acetic acid (IAA)**. Histograms of the algal cell population with and without 0.01 mM IAA addition are shown with the forward scatter **(A)** and Celltox green stain **(B)** after 8 d. The control in L1-Si medium with 1% ethanol is in black while the culture with IAA added is in red.

To determine a potential role for IAA produced by C cells of *E. huxleyi*, various concentrations of exogenous IAA were added to axenic cultures of both the C and N type strains in early log phase and then monitored for biomass (OD), cell morphology (microscopy and flow cytometry), chlorophyll and PSII health (PAM fluorometry), and membrane integrity (cell staining and flow cytometry; Figures 4–**6**). The C strain (CCMP3266) showed a small increase in potential quantum yield (F_v_/F_m_) with the addition of 0.1 mM exogenous IAA, while the N strain (CCMP2090) showed a greater increase in potential quantum yield with 0.1 mM of IAA (Figure 4). Lower concentrations of IAA did not affect the potential quantum yield (Figures 4A,B). An effect on potential quantum yield, which is an indicator of PSII health and overall photosynthetic performance, is consistent with the suggestion that IAA stimulates photosynthetic reactions in the chloroplast of plants (Tamás et al., 1972). Such a stimulating effect on photosynthesis has also been demonstrated in diatoms, which harbor bacterial symbionts producing IAA (Amin et al., 2015).

CCMP3266 was relatively unaffected physiologically even by high concentrations of IAA, showing a minor decrease in growth, with F_o_ (chlorophyll fluorescence) unaffected (Figure 4). However, IAA had a notable effect on the chlorophyll content and biomass of CCMP2090 from 8 to 16 d of the experiment (Figure 4). The more noticeable effect of IAA was at the transition from log to stationary phase, which is consistent with IAA having been shown to be most effective on aged plants samples, such as old samples of maize, rather than freshly cut samples (Evans and Cleland, 1985). Auxins are important regulators of the cell cycle (De Veylder et al., 2007), and have been shown to influence cell division in algae (Vance, 1987). The addition of IAA to diatoms, as well as the addition of IAA and IAA-like compounds (from kelp extracts) to unicellular green algae has been shown to increase biomass (Mazur et al., 2001; Li et al., 2007; Amin et al., 2015). However, elevated IAA can be toxic, with the concentration range for growth promotion being quite narrow (Fässler et al., 2010), which is consistent with its effect on *E. huxleyi* (Figure 4).

It is generally thought that auxin function in algae would most likely parallel its function in land plants (Bradley, 1991; Tarakhovskaya et al., 2007), and therefore macro-algae were more likely to be responsive to auxin addition due to its role in cell differentiation (Mazur et al., 2001). It has also been suggested that auxins would have no signaling role in microalgae, and that they are merely side-products of other metabolic functions in these organisms (Stirk et al., 2014). While there was no visual difference between CCMP3266 cultures with or without the addition of IAA, CCMP2090 showed a morphological switch when grown in the presence of IAA. Cells were noticeably larger when grown with IAA compared to the 1% EtOH solvent control (Figure 5), or with the addition of the IAA precursor L-tryptophan. This morphological change was confirmed by an increase in the forward scatter of the algal population taken at 8 d (Figure 6A). Cells were also stained with Celltox Green cytotoxicity assay, which binds to the DNA of cells with impaired membrane integrity, resulting in a fluorescent signal. Under this treatment, there was an increase in the fluorescence of cells treated with IAA, indicating an increase in membrane permeability and as such, an overall loss of cell membrane integrity (Figure 6B).

While auxins do not act directly on cell walls, stimulation of cell elongation and cell wall synthesis is one of its functions in plants (Evans and Cleland, 1985; Cleland, 2010), although high concentrations (~0.2 mM) of IAA can cause inhibition of cell wall synthesis (Baker and Ray, 1965). Auxins can induce acidification in cell walls and an increase in the cell membrane potential. This can be through the transport of the lipophilic form of IAA (IAAH) into the cell which affects the membrane potential as it then dissociates into the anionic form (IAA^−^) (Nelles, 1977; Cleland, 2010; Zhang and Van Duijn, 2014), which leads to the activation of plasma membrane H^+^-ATPases and potassium channels, inducing modifications to the cell wall and hyperpolarizing the membrane (Osakabe et al., 2013; Ng et al., 2015; Velasquez et al., 2016). This change in a cell membrane's pH gradient, and the subsequent modifications on the cell wall by the released enzymes, leads to the cleavage of load-bearing cell-wall crosslinks promoting the turgor pressure needed for cell expansion (Cleland, 2010; Velasquez et al., 2016). The hyperpolarization of the membrane potential has also been demonstrated in the macroalgae *Chara corallina* (Zhang et al., 2016). These changes in cell membrane structure may explain why the N cell type is more affected by IAA than the C cell type in terms of morphological differences, as coccoliths and acidic extracellular polysaccharide coat C type cells and perhaps provide protection from these effects of IAA. Although the coccoliths would not prevent the IAA from entering the cell, and the role of coccoliths has not been fully established, they have been suggested to provide additional strength due to their interweaving structure and may protect the integrity of the cell (Paasche, 2002). Alternatively, it is possible that the lack of a visual morphological effect in C cells as a result of exposure to exogenous IAA is a consequence of the capability of these cells to produce it endogenously.

### Role of IAA in bacterial-algal interactions

**Figure 7:**
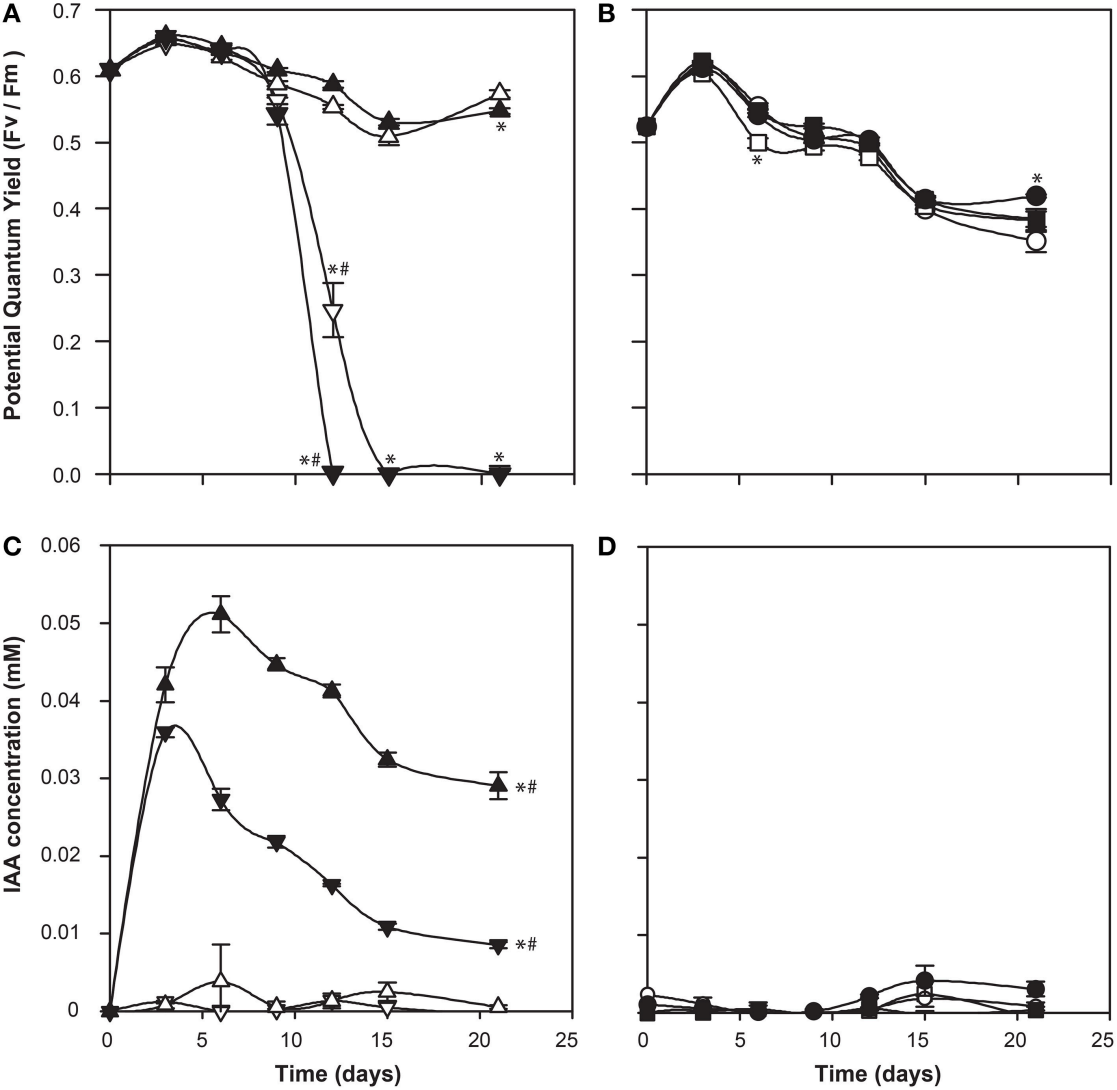
**Co-culture of *Ruegeria* sp. R11 with bald (CCMP2090) and coccolith bearing (CCMP3266) *E. huxleyi* strains**. The potential quantum yield of CCMP3266 **(A)** and CCMP2090 **(B)**, while IAA production (measured using the Salkowski reagent) for CCMP3266 **(C)**, and CCMP2090 **(D)** is shown. Triangles represent CCMP3266 alone, inverted triangles represent CCMP3266 co-cultured with R11, circles represent CCMP2090 alone and squares represent CCMP2090 co-cultured with R11. White shapes represent the control grown in L1-Si medium, while black indicates the addition of 0.1 mM tryptophan. Error bars represent ±1 SE. An asterisk (^*^) at a time point indicates that it is significantly different to the control and an asterisk at the end of the line indicates that the treatment is significantly different to the control. A hashtag (#) indicates the treatments are significantly different to the other treatment with a hashtag.

IAA has been shown to be an important bioactive molecule mediating bacterial-algal interactions between a diatom *Pseudo-nitzschia* and its bacterial community (Amin et al., 2015). In that system, the diatom up-regulated its tryptophan biosynthesis, resulting in its symbiotic bacterium *Sulfitobacter* sp. SA11 up-regulating its IAA biosynthesis genes. This relationship, where the alga produces tryptophan for the bacterium to convert into IAA, which in turn promotes algal growth, demonstrates a cross-kingdom relationship in which there is a metabolic and signaling exchange (Amin et al., 2015). The roseobacter *Ruegeria* sp. R11 has been shown to produce IAA (Fernandes et al., 2011), and its genome encodes the biosynthetic pathway (Figure 1 and Supplementary Table 1). Therefore, we postulated a role of IAA in the relationship between R11 and *E. huxleyi*, as R11 has recently been shown to be pathogenic to *E. huxleyi* CCMP3266, but not CCMP2090 (Mayers et al., 2016). However, the Salkowski reagent did not detect indolic compounds from R11 cultures grown in L1-Si medium or when co-cultured with *E. huxleyi*. Furthermore, the addition of L-tryptophan at a concentration that is not inhibitory to the growth of CCMP3266 did not stimulate the production of IAA in the bacterium (Figure 7). As R11 is known to make IAA, it may be that our methods are not sensitive enough to detect IAA production by R11, or that R11's biomass is not great enough to detect its IAA (since IAA was identified from 10 d cultures in ½YTSS medium which is much more nutrient rich than L1-Si medium and supports R11 cell density 1000 times higher) or that the host, *E. huxleyi*, drives IAA production in this partnership.

There was also little difference in *E. huxleyi's* potential quantum yield when co-cultured with R11 with or without the addition of L-tryptophan for CCMP2090 or CCMP3266. The only difference is that CCMP3266 died twice as fast from R11 infection when L-tryptophan was added to the co-culture (Figure 7). This could implicate tryptophan or IAA produced from tryptophan in R11 virulence or CCMP3266 host susceptibility, however further experiments are needed to decipher how tryptophan addition accelerates CCMP3266 death in co-culture with R11.

IAA concentration was expected to be elevated in the R11 co-cultures. However, less IAA was present compared to the alga grown alone for CCMP3266 supplemented with L-tryptophan and no IAA was detected from the R11-CCMP2090 co-culture. The source of the IAA produced in the R11-CCMP3266 co-culture supplemented with L-tryptophan is not known but this result is suggestive that R11 may divert L-tryptophan into alternate pathways. This is unlike the symbiotic relationship found by Amin et al. (2015) in *Pseudo-nitzschia*, where IAA plays a crucial role in the bacterial-host interaction. While R11 has been found in the same geographic area as *E. huxleyi*, they may not have a shared natural history. Their interaction is also different, pathogenic not symbiotic, and so IAA may play differing roles in symbiotic and pathogenic marine bacteria as it does with their terrestrial counterparts (Escobar and Dandekar, 2003; Vessey, 2003).

Regardless of its origin, we propose that IAA has evolved different signaling functions in haptophytes and diatoms. The data presented here suggest that IAA plays a role in cell-cell signaling between different cell types within an *E. huxleyi* population. In terrestrial plants and the red alga *G. dura*, IAA influences cell membrane permeability so as to direct cell maturation and differentiation. Therefore, IAA could be involved in similar processes in a unicellular alga, which while not having a multicellular form, has differentiated cell types within its population.

### Conclusion

IAA has been identified from a variety of photosynthetic organisms, including cyanobacteria (Sergeeva et al., 2002; Ahmed et al., 2010; Hussain et al., 2010), chlorophytes (Sztein et al., 2000; Mazur et al., 2001; Jirásková et al., 2009; Stirk et al., 2013), as well as some rhodophytes and brown algae (Le Bail et al., 2010; Mikami et al., 2015), but to our knowledge, this is the first time auxins have been identified from an axenic haptophyte culture. This raises further questions about the evolution of auxins, and their possible early role in cell-cell signaling between differentiated cell types within populations of unicellular organisms. Several lines of evidence suggest that the coccolith bearing (C) strain of *E. huxleyi* produces IAA, including: the putative presence of the pathway in the genome; the positive result of the Salkowski reagent when grown with L-tryptophan as well as the correct peak when analyzed with GC × GC-TOFMS; and the lack of this peak or Salkowski result when grown with D-tryptophan. The bald (N) strain did not test positive for IAA, but did show morphological changes in response to IAA. This suggests that not only does IAA play a role in the interaction between algae and their consortia of bacteria (Amin et al., 2015), but could play a role in the signaling between different cell types of algae themselves. These two cell types of *E. huxleyi* co-occur in *E. huxleyi* blooms, but have their highest population density at different times, with the N cell type increasing in proportion at the end of a bloom (Frada et al., 2012). It is therefore possible that IAA could play a role in cell-cell signaling in blooms, acting as a molecular signal throughout the bloom-bust cycle.

## Author contributions

LL and RC conceived the research. LL and JK carried out the bioinformatics analyses, AB, LL, and RC conducted the flow cytometry, LL, PM, and JH performed IAA analysis and LL conducted the growth experiments. LL, JK, HA, and RC drafted the manuscript. All authors have read and approved the manuscript.

## Funding

This work was supported by Natural Sciences and Engineering Research Council of Canada (grant 402105) to RC. JH and PM acknowledge the support of Genome Canada and Genome Alberta through grants to the Metabolomics Innovation Centre (TMIC).

### Conflict of interest statement

The authors declare that the research was conducted in the absence of any commercial or financial relationships that could be construed as a potential conflict of interest.
