# Supplementary material for: Indole-3-Acetic Acid Is Produced by Emiliania huxleyi Coccolith-Bearing Cells and Triggers a Physiological Response in Bald Cells
Source: Front Microbiol. 2016 Jun 8;7:828. doi: 10.3389/fmicb.2016.00828 (PMC4896954; doi:10.3389/fmicb.2016.00828)
Supplement: Supplementary file 1 [file Table1.PDF]

Tables

Table 1: Distribution of IAA biosynthesis genes in algal genomes

	YUCCA	AMI1	TAA1	CYP79B2	CYP79B3	AAO1	CYP71A13	TDC	MYR1	SUR1	SUR2	NIT1
**LAND PLANTS**												
Arabidopsis thaliana	AEE86075	Q9FR37	Q9S7N2	NP_195705	NP_179820	Q7G193	O49342	Q8RY79	P37702	O65782	Q9SIV0	AEE77887
**GREEN ALGAE**												
Ostreococcus sp RCC809[a]	-	-/+	-	-/+	-/+	-	-	-	-/+	-/+	+	-
Coccomyxa subellipsoidea[a]	-	+	-	-/+	-/+	+	-	+	-/+	-/+	-/+	+
Chlamydomonas reinhardtii[a]	-	+	-	-/+	-/+	+	-	+	-/+	-/+	-	-
**RED ALGAE**												
Cyanidioschyzon merolae[b]	-	-/+	-	-	-	-	-	-	-	-	+	-
Porphyridium purpureum[c]	-	-	-	-	-	-	-	-	-	-	-	-
Chondrus crispus[d]	-	-/+	-	-/+	-/+	+	-	-	-/+	-/+	+	-
**GLAUCOPHYTES**												
Cyanophora paradoxa[d]	-	-	-	-	-	-	-	-	-	-	-	-
**DIATOMS**												
Fragilariopsis cylindrus[a]	-	-	-	-	-	-	-	-	-	-	-	-
Phaeodactylum tricornutum[a]	-	-/+	-	-/+	-/+	+	-	-	-/+	-/+	+	-/+
Pseudo-nitzschia multiseries CLN-47[a]	+/-	-/+	-	-	-/+	+	-	-	-/+	-/+	+	-
**PELAGOPHYTE**												
Aureococcus anophagefferens[a]	-	-/+	-	-/+	-/+	+	-	-	-	-	+	-
**BROWN ALGAE**												
Ectocarpus siliculosus[d]	-	+	-	+	+	+	-	+	+	+	+	-
**EUSTIGMATOPHYTES**												

***Nannochloropsis oculata*[d]**	-	-	-	-	-	-	-	-	-	-	-	-
**CHRYSOPHYTES**												
***Ochromonas danica*[d]**	-	-	-	-	-	-	-	-	-	-	-	-
**DINOFLAGELLATES**												
***Symbiodinium minutum*[e]**	-	-	-	-	-	-	-	-	-	-	-	-
**CRYPTOPHYTES**												
***Guillardia theta*[a]**	+/-	-/+	-/+	-/+	-/+	+	-	-	-	-/+	-/+	-
***Hemiselmis andersenii*[d]**	-	-	-	-	-	-	-	-	-	-	-	-
HAPTOPHYTES												
***E. huxleyi CCMP1516*[d]**	+/-	-/+	-/+	-/+	-/+	+	-	+	-/+	-/+	+	+

[1]Footnote = Presence is determined initially by a bi-directional best hit (BBH) BLASTP hit with an E-value < $1x10^{-10}$ or less using the characterized land plant *A. thaliana* gene as a query, as well as correct functional prediction using ESG and orthology with the plant enzyme as determined by OrthoMCL. (+) indicates that it matches all three criteria, (-/+) indicates it is confirmed by ESG but not OrthoMCL, while (+/-) indicates confirmation by OrthoMCL but not ESG. The following databases were searched: JGI ([a]), http://merolae.biol.s.u-tokyo.ac.jp/blast/blast.html ([b]), http://cyanophora.rutgers.edu/porphyridium/ ([c]), NCBI ([d]), and OIST Marine Genomics Unit (http://marinegenomics.oist.jp/genomes/gallery/) ([e]). The abbreviations used for the enzyme can be described as follows: Tryptamine monooxygenase (YUCCA); Indole-3-acetamide hydrolase (AMI1); Tryptophan amino transferase (TAA1); Cytochrome P450s (CYP79B2 and CYP79B3); Indole-3-acetaldehyde oxidase (AAO1); Indole-acetaldoxime dehydratase (CYP71A13); Tryptophan decarboxylase (TDC); Myrosinase (MYR1); C-S lyase (SUR1); CYP83B1 (SUR2); and Nitralase (NIT1).

Table 2: GC×GC-TOFMS peak table

Sample ID	Name	R.T. (s) (1D*, 2D**)	Peak Area	Quant Masses	Similarity	Reverse	Signal to Noise (S/N) ratio
**IAA standard 2.8 mM**	3-Indoleacetic acid, trimethylsilyl ester	(1728, 1.020)	23810	73	816	846	812.56
	3-Indoleacetic acid, trimethylsilyl ester	(1774, 1.000)	218651	73	862	862	2760.90
**CCMP3266 0.1 mM L-tryptophan**	3-Indoleacetic acid, trimethylsilyl ester	(1728, 1.020)	1299	73	416	796	34.08
	3-Indoleacetic acid, trimethylsilyl ester	(1772, 0.990)	17418	73	765	793	639.58
**CCMP3266 0.1 mM D-tryptophan**	No peak found						
**CCMP3266 Control**	No peak found						
**CCMP2090 0.1 mM L-tryptophan**	No peak found						
**CCMP2090 0.1 mM D-tryptophan**	No peak found						
**CCMP2090 Control**	No peak found						
**L1-Si Medium (seawater)**	Unknown	(1772, 0.990)	290	73	NS#	NS	10.80

*1D = 1st Dimension, **2D = 2nd Dimension, #NS = Not searchable
