# Supplementary material for: Indole-3-Acetic Acid Is Produced by Emiliania huxleyi Coccolith-Bearing Cells and Triggers a Physiological Response in Bald Cells
Source: Front Microbiol. 2016 Jun 8;7:828. doi: 10.3389/fmicb.2016.00828 (PMC4896954; doi:10.3389/fmicb.2016.00828)
Supplement: Supplementary file 2 [file DataSheet1.pdf]

Supplementary Material

Indole-3-acetic acid is produced by *Emiliania huxleyi* coccolith-bearing cells and triggers a physiological response in bald cells

Leen Labeeuw, Joleen Khey, Anna R. Bramucci, Harjot Atwal, Paulina de la Mata, James Harynuk, and Rebecca J. Case*

*** Correspondence:** Rebecca J. Case: rcase@ualberta.ca

Note about the supplementary tables:

Presence is determined initially by a bi-directional best hit (BBH) BLASTP hit with an E-value < $1x10^{-10}$ or less, with the correct functional prediction, using the characterized bacterial genes as queries for Suppl. Table 1 (p2), and plant *A. thaliana* gene as a query for Suppl. Table 2 (p10), followed by ESG functional prediction and OrthoMCL orthology.

(+) indicates that it is matches all three criteria, (-/+) indicates it is absent in OrthoMCL, present in ESG, while (+/-) indicates presence in OrthoMCL, absence in ESG. Presence in OrthoMCL was determined if it had the same group as the seed sequence. Presence in ESG was determined if it fell in the same confidence interval as the seed sequence, where >70% indicates very high confidence, >40% indicates high confidence, >=30% indicates moderate confidence, and below 30% indicates below moderate confidence.

The following databases were searched: JGI (http://genome.jgi.doe.gov/) ([a]), http://merolae.biol.s.u-tokyo.ac.jp/blast/blast.html ([b]), http://cyanophora.rutgers.edu/porphyridium/ ([c]), NCBI (http://www.ncbi.nlm.nih.gov/) ([d]), and OIST Marine Genomics Unit (http://marinegenomics.oist.jp/genomes/gallery/) ([e]). The abbreviations used for the enzyme can be described as follows: Tryptamine monooxygenase (YUCCA); Indole-3-acetamide hydrolase (AMI1); Tryptophan amino transferase (TAA1); Cytochrome P450s (CYP79B2 and CYP79B3); Indole-3-acetaldehyde oxidase (AAO1); Indole-acetaldoxime dehydratase (CYP71A13); Tyrosine decarboxy lase (TDC); Myrosinase (MYR1); C-S lyase (SUR1); CYP83B1 (SUR2); and Nitralase (NIT1).

Supplementary Table 1: Distribution of bacterial IAA biosynthesis genes in roseobacter and algal genomes

	Enzyme	Nitrile Hydratase		Tryptophan mono-oxygenase	Indole-3-acetamide hydrolase	Indole-3-pyruvate decarboxylase	Indole-3-acetaldehyde dehydrogenase	Tryptophan decarboxylase	Copper amine/ tyramine oxidase
TERRESTRIAL									
Agrobacterium tumefaciens C58 [d]	Accession #			AAD30489	AAD30488				
Agrobacterium radiobacter K84 [d]	Accession #	ACM26968	ACM26967						
Pseudomonas aeruginoasa PA7 [d]	Accession #			ABR84797					
Enterobacter cloacae [d]	Accession #					P23234			
Paenibacillus polymyxa [d]	Accession #					ABV24338			
Ustilago maydis FB1 [d]	Accession #						AAC49575		
Catharanthus roseu s [d]	Accession #							CAA47898	
Enterobacter aerogenes [d]	Accession #								P49250
Klebsiella pneumoniae [d]	Accession #	AIE03299	AIE03300						
	**Ortho MCL**	OG5_ 194743	NO_ GROUP	OG5_130946	OG5_ 175845	OG5_129158, OG5_126899	OG5_126638	OG5_127644	OG5_ 127171
	**ESG Molecular Function terms**	indole-3-acetonitrile nitrile hydratase activity	indole-3-acetonitrile nitrile hydratase activity	L-amino-acid oxidase activity	glutaminyl-tRNA synthase (glutamine-hydrolyzing) activity,	pyruvate decarboxylase activity	oxidoreductase activity,	aromatic-L-amino-acid decarboxylase activity	copper ion binding
	**ESG Confidence**	89.60%	90.40%	100.00%	93%	53.80%	38.40%	43.50%	50.60%
**ROSEOBACTER**									
Ruegeria sp. R11 [d]	**Accession #**	EEB72184	EEB70836	-	WP_008560074	EEB71015	EEB70673	WP_008558890	-
	**E-value**	2.00E-86	2.00E-47	-	8.00E-33	3.00E-51	7.00E-113	4.00E-105	-
	**Reciprocal e-value**	3.00E-84	7.00E-42	-	0.00E+00	2.00E-57	2.00E-112	1.00E-100	-
	**OrthoMCL**	OG5_ 194743	NO_GROUP		OG5_127466	OG5_126899	OG5_131868	OG5_129065	
	**ESG Confidence**	89.40%	90.10%		92.00%	0.00%	48.10%	47.70%	-
	**Presence**	+	+	-	-/+	+/-	-/+	-/+	-

Dinoroseobacter shibae DFL 12 [d]	**Accession #**	WP_012178521	WP_012178520	-	WP_012179446	WP_012178451	WP_012177768	-	-
	**E-value**	3.00E-59	2.00E-10	-	8.00E-34	0.00E+00	2.00E-122	-	-
	**Reciprocal e-value**	5.00E-57	5.00E-09	-	1.00E-41	0.00E+00	2.00E-132	-	-
	**OrthoMCL**	OG5_194743	NO_GROUP	OG5_130946	OG5_127466	OG5_126899	OG5_126638		
	**ESG Confidence**	88.80%	87.50%	-	88.00%	0.00%	32.60%		-
	**Presence**	+	+	-	-/+	+/-	+	-	-
Jannaschia sp. CCS1 [d]	**Accession #**	-	-	-	ABD56894	WP_044006611	WP_011456626	WP_011456620	-
	**E-value**	-	-	-	2.00E-54	0.00E+00	1.00E-119	5.00E-88	-
	**Reciprocal e-value**	-	-	-	4.00E-58	0.00E+00	2.00E-121	2.00E-90	-
	**OrthoMCL**				OG5_175845	OG5_126899	OG5_131868	OG5_129065	
	**ESG Confidence**				100.00%	0.00%	44.60%	46.90%	
	**Presence**	+	+	-	+	+/-	-/+	-/+	-
Phaeobacter gallaeciensis 2.10 [d]	**Accession #**	AFO88179	AFO88178	-	WP_014875196	WP_014875766	WP_014873197	WP_014873282	-
	**E-value**	5.00E-91	3.00E-42	-	1.00E-31	1.00E-50	3.00E-118	3.00E-108	-
	**Reciprocal e-value**	1.00E-88	3.00E-37	-	0.00E+00	1.00E-56	5.00E-122	1.00E-103	-
	**OrthoMCL**	OG5_194743	NO_GROUP		OG5_127466	OG5_126899	OG5_131868	OG5_129065	
	**ESG Confidence**	89.40%	89.90%		93.50%	0.00%	43.50%	47.10%	-
	**Presence**	+	+	-	-/+	+/-	-/+	-/+	-
Phaeobacter gallaeciensis DSM17395 (BS107) [d]	**Accession #**	AFO92073	WP_014880670	-	WP_014880496	WP_014880356	WP_014881318	WP_014881252	-
	**E-value**	1.00E-89	5.00E-57	-	3.00E-32	0.00E+00	2.00E-119	1.00E-107	-
	**Reciprocal e-value**	3.00E-87	3.00E-38	-	0.00E+00	0.00E+00	4.00E-123	4.00E-103	-
	**OrthoMCL**	OG5_194743	NO_GROUP		OG5_127466	OG5_126899	OG5_131868	OG5_129065	
	**ESG Confidence**	89.40%	89.40%		94.00%	0.00%	42.00%	40.70%	-
	**Presence**	+	+	-	-/+	+/-	-/+	-/+	-

Roseobacter denitrificans OCh 114 [d]	**Accession #**	WP_ 011568135	WP_ 011568136	WP_ 011567748	WP_ 011568221	WP_ 011568956	WP_011568243	-	-
	**E-value**	1.00E-91	2.00E-53	6.00E-94	5.00E-34	0.00E+00	4.00E-115	-	-
	**Reciprocal e-value**	3.00E-89	1.00E-39	1.00E-47	4.00E-42	0.00E+00	2.00E-126	-	-
	**OrthoMCL**	OG5_ 194743	NO_ GROUP	OG5_130946	OG5_ 127466	OG5_126899	OG5_131437		
	**ESG Confidence**	89.30%	89.30%	100.00%	93.00%	0.00%	36_0%		-
	**Presence**	+	+	+	-/+	+/-	-/+	-	-
Ruegeria pomeroyi DSS-3 [d]	**Accession #**	WP_ 011047053	WP_ 011047054	-	WP_ 011047226	WP_ 011048283	WP_011241895	WP_ 011049365	-
	**E-value**	1.00E-91	1.00E-48	-	2.00E-100	0.00E+00	7.00E-139	3.00E-112	-
	**Reciprocal e-value**	3.00E-89	4.00E-41	-	3.00E-104	0.00E+00	7.00E-141	7.00E-107	-
	**OrthoMCL**	OG5_ 194743	NO_ GROUP		OG5_ 175845	OG5_126899	OG5_168711	OG5_129065	
	**ESG Confidence**	89.40%	89.40%		96.60%	0.00%	36.00%	41.30%	-
	**Presence**	+	+	-	+	+/-	-/+	-/+	-
Ruegeria sp. TM1040 [d]	**Accession #**	WP_ 011539297	WP_ 011539296	-	WP_ 011539245	WP_ 011538220	WP_011537227	WP_ 011537081	-
	**E-value**	1.00E-91	1.00E-48	-	2.00E-100	0.00E+00	7.00E-139	3.00E-112	-
	**Reciprocal e-value**	2.00E-83	6.00E-36	-	0.00E+00	0.00E+00	6.00E-131	4.00E-99	-
	**OrthoMCL**	OG5_ 194743	NO_ GROUP		OG5_ 127466	OG5_126899	OG5_126638	OG5_129065	
	**ESG Confidence**	89.40%	89.40%		94.00%	0.00%	32.10%	47.10%	-
	**Presence**	+	+	-	-/+	+/-	+	-/+	-
**LAND PLANT**									
Arabidopsis thaliana [d]	**Accession #**	none	none	none	AAF73891_	NP_200307	NP_190383	NP_849999	NP_ 176469
	**E-value**	-	-	-	7.00E-31	6.00E-81	5.00E-180	0	1.00E-96
	**Reciprocal e-value**	-	-	-	8.00E-33	5.00E-81	4.00E-82	0	2.00E-97
	**OrthoMCL**	-	-	-	OG5_ 132924	OG5_129158	OG5_126638	OG5_129065	OG5_ 127171
	**ESG Confidence**	-	-	-	96.00%	87.50%	25.50%	52.70%	62.40%
	**Presence**	-	-	-	-/+	+	+/-	-/+	+

**GREEN ALGAE**									
Ostreococcus sp RCC809 [a]	**Accession #**	none	none	none	29365	41388	58690	28940	none
	**E-value**	-	-	-	3.48E-26	8.28E-145	9.97E-61	4.68E-20	-
	**Reciprocal e-value**	-	-	-	5.00E-42	6.00E-20	2.00E-71	3.00E-31	-
	**OrthoMCL**	-	-	-	OG5_ 127466	OG5_126899	OG5_126638	OG5_127644	-
	**ESG Confidence**	-	-	-	88.50%	0.00%	43.50%	0.00%	-
	**Presence**	-	-	-	-/+	+/-	+	+/-	-
Coccomyxa subellipsoidea [a]	**Accession #**	none	none	none	XP_ 005651884	XP_ 005645798	XP_ 005642981	XP_ 005646768	XP_ 005649283
	**E-value**	-	-	-	7.00E-59	3.00E-175	0	4.00E-158	4.00E-83
	**Reciprocal e-value**	-	-	-	6.00E-61	2.00E-98	2.00E-85	4.00E-155	7.00E-84
	**OrthoMCL**	-	-	-	OG5_ 175845	OG5_129158	OG5_126638	OG5_129065	OG5_ 127171
	**ESG Confidence**	-	-	-	100.00%	59.40%	46.60%	52.70%	89.20%
	**Presence**	-	-	-	+	+	+	-/+	+
Chlamydomonas reinhardtii [a]	**Accession #**	none	none	none	XP_ 001690552	AAB88292	XP_001690955	XP_ 001690025	XP_ 001697595
	**E-value**	-	-	-	6.00E-31	5.00E-174	3.00E-180	8.00E-133	6.00E-70
	**Reciprocal e-value**	-	-	-	1.00E-40	4.00E-92	8.00E-97	9.00E-140	8.00E-81
	**OrthoMCL**	-	-	-	OG5_ 127466	OG5_129158	OG5_126638	OG5_129065	OG5_ 127171
	**ESG Confidence**	-	-	-	87.00%	59.20%	27.10%	40.10%	80.90%
	**Presence**	-	-	-	-/+	+	+/-	-/+	+
**RED ALGAE**									
Cyanidioschyzon merolae [b]	**Accession #**	none	none	none	XP_ 005537175	NP_849033	XP_005538376	none	XP_ 005538693
	**E-value**	-	-	-	7.00E-27	1.00E-166	1.00E-94	-	9.00E-89
	**Reciprocal e-value**	-	-	-	5.00E-38	1.00E-25	8.00E-92	-	1.00E-89
	**OrthoMCL**	-	-	-	OG5_ 127466	OG5_126899	OG5_127004	-	OG5_ 127171
	**ESG Confidence**	-	-	-	96.50%	0.00%	30.50%	-	67.60%
	**Presence**	-	-	-	-/+	+/-	-/+	-	+

Porphyridium purpureum [c]	**Accession #**	none	none	none	none	YP_ 008965627	none	none	none
	**E-value**	-	-	-	-	6.00E-175	-	-	-
	**Reciprocal e-value**	-	-	-	-	2.00E-28	-	-	-
	**OrthoMCL**	-	-	-	-	OG5_126899	-	-	-
	**ESG Confidence**	-	-	-	-	0.00%	-	-	-
	**Presence**	-	-	-	-	+/-	-	-	-
Chondrus crispus [d]	**Accession #**	none	none	none	XP_ 005710130	YP_ 007627349	XP_005718776	none	none
	**E-value**	-	-	-	3.00E-30	5.00E-178	4.00E-63	-	-
	**Reciprocal e-value**	-	-	-	7.00E-40	2.00E-22	1.00E-62	-	-
	**OrthoMCL**	-	-	-	OG5_ 127466	OG5_126899	OG5_127004	-	-
	**ESG Confidence**	-	-	-	93.50%	0.00%	40.40%	-	-
	**Presence**	-	-	-	-/+	+/-	-/+	-	-
**GLAUCOPHYTE**					-				
Cyanophora paradoxa [d]	**Accession #**	none	none	none	none	none	none	none	none
	**E-value**	-	-	-	-	-	-	-	-
	**Reciprocal e-value**	-	-	-	-	-	-	-	-
	**OrthoMCL**	-	-	-	-	-	-	-	-
	**ESG Confidence**	-	-	-	-	-	-	-	-
	**Presence**	-	-	-	-	-	-	-	-
**DIATOMS**									
Fragilariopsis cylindrus [a]	**Accession #**	none	none	none	none	none	none	none	none
	**E-value**	-	-	-	-	-	-	-	-
	**Reciprocal e-value**	-	-	-	-	-	-	-	-
	**OrthoMCL**	-	-	-	-	-	-	-	-
	**ESG Confidence**	-	-	-	-	-	-	-	-
	**Presence**	-	-	-	-	-	-	-	-

Phaeodactylum tricornutum [a]	**Accession #**	none	none	none	XP_ 002180426	XP_ 002181443	XP_002185692	none	XP_ 002180619
	**E-value**	-	-	-	1.00E-16	5.00E-59	1.00E-72	-	9.00E-60
	**Reciprocal e-value**	-	-	-	1.00E-18	1.00E-05	5.00E-66	-	9.00E-61
	**OrthoMCL**	-	-	-	OG5_ 127783	OG5_126899	OG5_127004	-	OG5_ 127171
	**ESG Confidence**	-	-	-	100.00%	0.00%	32.60%	-	80.90%
	**Presence**	-	-	-	-/+	+/-	-/+	-	+
Pseudo-nitzschia multiseries CLN-47 [a]	**Accession #**	55399	none	none	228151	237689	298461	none	243692
	**E-value**	3.98E-41	-	-	1.76E-12	1.84E-67	2.07E-68	-	4.59E-45
	**Reciprocal e-value**	2.00E-43	-	-	2.00E-11	1.00E-09	1.00E-57	-	2.00E-62
	**OrthoMCL**	OG5_ 194743	-	-	OG5_ 127466	OG5_126899	OG5_137356	-	OG5_ 127171
	**ESG Confidence**	90.20%	-	-	100.00%	0.00%	39.60%	-	84.00%
	**Presence**	+	-	-	-/+	+/-	-/+	-	+
**PELAGOPHYTE**									
Aureococcus anophagefferens [a]	**Accession #**	XP_ 009036233	none	none	XP_ 009032301	YP_ 003002081	XP_009041380	XP_ 009042616	none
	**E-value**	9.00E-41	-	-	6.00E-31	4.00E-20	4.00E-145	1.00E-13	-
	**Reciprocal e-value**	7.00E-43	-	-	7.00E-39	3.00E-20	4.00E-75	5.00E-14	-
	**OrthoMCL**	OG5_ 194743	-	-	OG5_ 175845	OG5_126899	OG5_126638	OG5_127644	-
	**ESG Confidence**	89.30%	-	-	98.00%	0.00%	34.60%	0.00%	-
	**Presence**	+	-	-	+	+/-	+	+/-	-
**BROWN ALGAE**									
Ectocarpus siliculosus [d]	**Accession #**	none	none	none	CBN78043	YP_ 003289152	CBJ33263	CBN75443	CBN75262
	**E-value**	-	-	-	3.00E-24	6.00E-19	6.00E-95	4.00E-97	2.00E-84
	**Reciprocal e-value**	-	-	-	5.00E-36	9.00E-19	2.00E-59	8.00E-101	3.00E-85
	**OrthoMCL**	-	-	-	OG5_ 127466	OG5_126899	OG5_137356	OG5_129065	OG5_ 127171
	**ESG Confidence**	-	-	-	100.00%	0.00%	37.00%	46.80%	80.60%
	**Presence**	-	-	-	-/+	+/-	-/+	-/+	+

**EUSTIGMATOPHYTE**									
Nannochloropsis oculata [d]	**Accession #**	none	none	none	none	AHX25385	none	none	none
	**E-value**	-	-	-	-	5.00E-22	-	-	-
	**Reciprocal e-value**	-	-	-	-	4.00E-22	-	-	-
	**OrthoMCL**	-	-	-	-	OG5_126899	-	-	-
	**ESG Confidence**	-	-	-	-	0.00%	-	-	-
	**Presence**	-	-	-	-	+/-	-	-	-
**CHRYSOPHYTE**									
Ochromonas danica [d]	**Accession #**	none	none	none	none	none	none	none	none
	**E-value**	-	-	-	-	-	-	-	-
	**Reciprocal e-value**	-	-	-	-	-	-	-	-
	**OrthoMCL**	-	-	-	-	-	-	-	-
	**ESG Confidence**	-	-	-	-	-	-	-	-
	**Presence**	-	-	-	-	-	-	-	-
**DINOFLAGELLATES**									
Symbiodinium minutum [e]	**Accession #**	none	none	none	none	none	none	none	none
	**E-value**	-	-	-	-	-	-	-	-
	**Reciprocal e-value**	-	-	-	-	-	-	-	-
	**OrthoMCL**	-	-	-	-	-	-	-	-
	**ESG Confidence**	-	-	-	-	-	-	-	-
	**Presence**	-	-	-	-	-	-	-	-
**CRYPTOPHYTE**									
Guillardia theta [a]	**Accession #**	none	none	none	XP_005837094	NP_050806	XP_005835269	XP_005821099	none
	**E-value**	-	-	-	1.00E-15	3.00E-20	4.00E-175	1.00E-31	-
	**Reciprocal e-value**	-	-	-	6.00E-18	2.00E-20	7.00E-84	3.00E-30	-
	**OrthoMCL**	-	-	-	OG5_132214	OG5_126899	OG5_126638	OG5_127644	-
	**ESG Confidence**	-	-	-	100.00%	0.00%	44.60%	0.00%	-
	**Presence**	-	-	-	-/+	+/-	+	+/-	-

Hemiselmis andersenii [d]	**Accession #**	none	none	none	none	none	none	none	none
	**E-value**	-	-	-	-	-	-	-	-
	**Reciprocal e-value**	-	-	-	-	-	-	-	-
	**OrthoMCL**	-	-	-	-	-	-	-	-
	**ESG Confidence**	-	-	-	-	-	-	-	-
	**Presence**	-	-	-	-	-	-	-	-
**HAPTOPHYTE**									
Emiliania huxleyi CCMP1516 [d]	**Accession #**	XP_ 005756629	XP_ 005756628	none	XP_ 005537175	XP_ 005756827	XP_005779034	XP_ 005776490	XP_ 005767244
	**E-value**	2.00E-27	1.00E-11	-	7.00E-27	8.00E-99	3.00E-123	2.00E-32	2.00E-19
	**Reciprocal e-value**	2.00E-25	-	-	2.00E-30	3.00E-25	5.00E-76	1.00E-32	4.00E-20
	**OrthoMCL**	OG5_ 194743	OG5_ 194743	-	OG5_ 127466	OG5_126899	OG5_126638	OG5_129065	OG5_ 127171
	**ESG Confidence**	88.00%	90.40%	-	95.50%	0.00%	29.00%	47.10%	76.80%
	**Presence**	+	-/+	-	-/+	+/-	+/-	-/+	+

Supplementary Table 2: Distribution of IAA plant biosynthesis genes in algal and roseobacter genomes

	Code	YUCCA	AMI1	TAA1	CYP79B2	CYP79B3	AAO1	CYP71A13	TDC	MYR1	SUR1	SUR2	NIT1
**LAND PLANT**													
Arabidopsis thaliana [d]	**Accession #**	AEE86075	Q9FR37	Q9S7N2	NP_195705	NP_179820	Q7G193	O49342	Q8RY79	P37702	O65782	Q9SIV0	AEE77887
	**OrthoMCL group**	OG5_ 129359	OG5_ 149946	OG5_ 189781	OG5_ 140471	OG5_ 140471	OG5_ 127252	OG5_146283	OG5_ 129065	OG5_ 164273	OG5_ 126582	OG5_ 128985	OG5_ 131865
	**ESG Molecular Function terms**	N,N-dimethylaniline monooxygenase activity,	glutaminyl-tRNA synthase (glutamine-hydrolyzing) activity	L-phenylalanine:2-oxoglutarate amino-transferase activity	monooxygenase activity	monooxygenase activity	oxidoreductase activity	oxidoreductase activity	aromatic-L-amino-acid decarboxylase activity	hydrolase activity, hydrolyzing O-glycosyl compounds	oxidoreductase activity	L-phenylalanine: 2-oxoglutarate amino-transferase activity	nitrilase activity
	**ESG Confidence**	0.00%	100.00%	48.00%	42.70%	53.20%	100.00%	0.00%	52.70%	71.40%	30.50%	33.00%,	31.80%
**GREEN ALGAE**													
Ostreococcus sp RCC809 [a]	**Accession #**	39261	38981	none	40254	40254	none	58872	28940	38969	40254	27915	none
	**E-value**	4.54E-06	2.03E-61	-	1.08E-14	1.01E-16	-	1_0E-16	8.93E-24	1.53E-43	7.80E-25	1_7E-72	-
	**Reciprocal e-value**	4.00E-14	5.00E-85	-	0	0	-	4.00E-18	2.00E-37	2.00E-109	0.00E+00	2.00E-75	-
	**OrthoMCL**	-	OG5_ 150132	-	OG5_ 126554	OG5_ 126554	-	OG5_134261	OG5_ 127644	OG5_ 126690	OG5_ 126554	OG5_ 128985	-
	**ESG Confidence**	-	96.50%	-	49.60%	49.60%	-	36.10%	0.00%	59.50%	47.60%	55.50%	-
	**Presence**	-	-/+	-	-/+	-/+	-	-	-	-/+	-/+	+	-
Coccomyxa subellipsoidea [a]	**Accession #**	XP_ 005652161	XP_ 005647194	none	XP_ 005648231	XP_ 005648231	XP_ 005646284	XP_ 005652354	XP_ 005646768	XP_ 005643327	XP_ 005648231	XP_ 005650249	XP_ 005646017
	**E-value**	1.00E-36	2.00E-100	-	8.00E-19	2.00E-19	9.00E-164	2.00E-25	2.00E-170	3.00E-101	4.00E-30	9.00E-19	3.00E-45
	**Reciprocal e-value**	2.00E-37	7.00E-101	-	0	0	0	0	3.00E-171	3.00E-152	0	1.00E-19	4.00E-46
	**OrthoMCL**	OG5_ 126653	OG5_ 149946	-	OG5_ 134261	OG5_ 134261	OG5_ 127252	OG5_126554	OG5_ 129065	OG5_ 126690	OG5_ 134261	OG5_ 135328	OG5_ 131865
	**ESG Confidence**	73.40%	100.00%	-	53.00%	53.00%	100.00%	43.80%	47.10%	70.00%	44.40%	100.00%	30.10%
	**Presence**	-	+	-	-/+	-/+	+	-	+	-/+	-/+	-/+	+

Chlamydomonas reinhardtii [a]	**Accession #**	XP_ 005779538	XP_ 001698766	none	XP_ 001698892	ABQ59243	XP_ 001694090	XP_ 001700492	XP_ 00169005	XP_ 001700848	XP_ 001698892	AAB01685	XP_ 001692986
	**E-value**	1.00E-14	2.00E-50	-	2.00E-16	2.00E-17	8.00E-164	1.00E-28	2.00E-150	1.00E-18	2.00E-23	8.00E-15	5.00E-07
	**Reciprocal e-value**	3.00E-15	8.00E-61	-	2.00E-163	0.00E+00	2.00E-151	0.00E+00	9.00E-163	3.00E-33	2.00E-163	2.00E-15	2.00E-09
	**OrthoMCL**	OG5_ 126653	OG5_ 149946	-	OG5_ 126554	OG5_ 134261	OG5_ 127252	OG5_126554	OG5_ 129065	OG5_ 126690	OG5_ 126554	OG5_ 127112	n/a
	**ESG Confidence**	77.90%	95.50%	-	45.00%	44.80%	100.00%	38.30%	40_0%	64.60%	40_0%	0.00%	n/a
	**Presence**	-	+	-	-/+	-/+	+	-	+	-/+	-/+	-	-
**RED ALGAE**													
Cyanidioschyzon merolae [b]	**Accession #**	none	XP_ 005537175	none	none	XP_ 005534988	none	XP_ 005535456	none	none	XP_ 005535456	XP_ 005539487	XP_ 005538174
	**E-value**	-	4.00E-21	-	-	1.00E-09	-	1.00E-08	-	-	8.00E-09	2.00E-65	8.00E-08
	**Reciprocal e-value**	-	1.00E-126	-	-	4.00E-123	-	1.00E-16	-	-	1.00E-16	7.00E-66	1.00E-86
	**OrthoMCL**	-	OG5_ 127466	-	-	n/a	-	n/a	-	-	n/a	OG5_ 128985	n/a
	**ESG Confidence**	-	96.50%	-	-	n/a	-	n/a	-	-	n/a	68.30%	n/a
	**Presence**	-	-/+	-	-	-	-	-	-	-	-	+	-
Porphyridium purpureum [c]	**Accession #**	none	none	none	none	none	none	none	none	none	none	none	none
	**E-value**	-	-	-	-	-	-	-	-	-	-	-	-
	**Reciprocal e-value**	-	-	-	-	-	-	-	-	-	-	-	-
	**OrthoMCL**	-	-	-	-	-	-	-	-	-	-	-	-
	**ESG Confidence**	-	-	-	-	-	-	-	-	-	-	-	-
	**Presence**	-	-	-	-	-	-	-	-	-	-	-	-
Chondrus crispus [d]	**Accession #**	none	XP_ 005710130	none	XP_ 005713400	XP_ 005713400	XP_ 005718689	XP_ 005715170	none	XP_ 005715104	XP_ 005715216	XP_ 005713817	none
	**E-value**	-	7.00E-21	-	2.00E-19	5.00E-18	5.00E-168	1.00E-21	-	4.00E-94	9.00E-23	3.00E-70	-
	**Reciprocal e-value**	-	6.00E-139	-	1.00E-46	1.00E-46	0.00E+00	2.00E-39	-	1.00E-132	0.00E+00	1.00E-72	-
	**OrthoMCL**	-	OG5_ 127466	-	OG5_ 126554	OG5_ 126554	OG5_ 127252	OG5_126554	-	OG5_ 126690	OG5_ 126554	OG5_ 128985	-
	**ESG Confidence**	-	93.50%	-	60.10%	60.10%	99.50%	30.80%	-	44.50%	32.90%	50.60%	-
	**Presence**	-	-/+	-	-/+	-/+	+	-	-	-/+	-/+	+	-

**GLAUCOPHYTE**													
Cyanophora paradoxa [d]	**Accession #**	none	none	none	none	none	none	none	none	none	none	none	none
	**E-value**	-	-	-	-	-	-	-	-	-	-	-	-
	**Reciprocal e-value**	-	-	-	-	-	-	-	-	-	-	-	-
	**OrthoMCL**	-	-	-	-	-	-	-	-	-	-	-	-
	**ESG Confidence**	-	-	-	-	-	-	-	-	-	-	-	-
	**Presence**	-	-	-	-	-	-	-	-	-	-	-	-
**DIATOMS**													
Fragilariopsis cylindrus [a]	**Accession #**	none	none	none	none	none	none	none	none	none	none	none	none
	**E-value**	-	-	-	-	-	-	-	-	-	-	-	-
	**Reciprocal e-value**	-	-	-	-	-	-	-	-	-	-	-	-
	**OrthoMCL**	-	-	-	-	-	-	-	-	-	-	-	-
	**ESG Confidence**	-	-	-	-	-	-	-	-	-	-	-	-
	**Presence**	-	-	-	-	-	-	-	-	-	-	-	-
Phaeodactylum tricornutum [a]	**Accession #**	none	XP_ 002180426	none	XP_ 002185035	XP_ 002185035	XP_ 002184143	XP_ 002178044	none	XP_ 002185317	XP_ 002185035	XP_ 002186145	XP_ 002183613
	**E-value**	-	4.00E-13	-	2.00E-11	9.00E-11	7.00E-132	6.00E-08	-	6.00E-104	5.00E-22	1.00E-51	4.00E-17
	**Reciprocal e-value**	-	5.00E-21	-	0	0	8.00E-138	2.00E-17	-	1.00E-140	0	9.00E-54	2.00E-97
	**OrthoMCL**	-	OG5_ 127783	-	OG5_ 126554	OG5_ 126554	OG5_ 127252	n/a	-	OG5_ 126690	OG5_ 126554	OG5_ 128985	OG5_ 126651
	**ESG Confidence**	-	100.00%	-	44.00%	44.00%	100.00%	n/a	-	74_0%	39_0%	99.00%	0.00%
	**Presence**	-	-/+	-	-/+	-/+	+	-	-	-/+	-/+	+	-/+
Pseudo-nitzschia multiseries CLN-47 [a]	**Accession #**	26050	54595	none	7819	8596	36829	1342	none	41676	7819	13947	289105
	**E-value**	1.47E-25	2.64E-13	-	8.47E-08	5.72E-11	1.92E-84	5_2E-12	-	2.56E-90	1.74E-18	1.30E-37	1.73E-06
	**Reciprocal e-value**	1.00E-25	7.00E-46	-	1.00E-36	4.00E-21	5.00E-120	6.00E-165	-	2.00E-135	1.00E-36	1.00E-55	1.00E-12
	**OrthoMCL**	OG5_ 129359	OG5_ 127466	-	n/a	OG5_ 127298	OG5_ 127252	OG5_126554	-	OG5_ 126690	OG5_ 169987	OG5_ 128985	n/a
	**ESG Confidence**	69.40%	100.00%	-	n/a	51.50%	100.00%	31.30%	-	48.90%	80.00%	64.10%	n/a
	**Presence**	+/-	-/+	-	-	-/+	+	-	-	-/+	-/+	+	-

**PELAGOPHYTE**													
Aureococcus anophagefferens [a]	**Accession #**	XP_ 009036936	XP_ 009039682	none	XP_ 009039697	XP_ 009039697	XP_ 009034984	XP_ 009032102	none	XP_ 009035359	XP_ 009032928	XP_ 009033544	XP_ 009039155
	**E-value**	3.00E-16	2.00E-18	-	2.00E-12	1.00E-12	5.00E-126	3.00E-15	-	2.00E-78	2.00E-07	3.00E-30	3.00E-11
	**Reciprocal e-value**	8.00E-17	7.00E-47	-	1.00E-28	1.00E-28	4.00E-148	4.00E-24	-	4.00E-129	2.00E-164	1.00E-31	7.00E-65
	**OrthoMCL**	OG5_ 126653	OG5_ 132924	-	OG5_ 126554	OG5_ 126554	OG5_ 127252	OG5_126554	-	OG5_ 126690	OG5_ 126554	OG5_ 128985	OG5_ 126651
	**ESG Confidence**	83.50%	100.00%	-	47.50%	47.50%	100.00%	33.40%	-	52.50%	n/a	100.00%	0.00%
	**Presence**	-	-/+	-	-/+	-/+	+	-	-	-	-	+	-
**BROWN ALGAE**													
Ectocarpus siliculosus [d]	**Accession #**	CBJ32825	CBN78043	none	CBJ27249	CBJ27249	CBJ27195	CBN74955	CBN 75443	CBN79091	CBJ27248	CBJ25743	CBN74568
	**E-value**	3.00E-15	2.00E-14	-	1.00E-13	4.00E-13	9.00E-119	8.00E-06	2.00E-109	7.00E-92	1.00E-24	6.00E-21	1.00E-10
	**Reciprocal e-value**	5.00E-17	5.00E-93	-	0.00E+00	0.00E+00	8.00E-121	1.00E-07	2.00E-117	1.00E-130	0.00E+00	3.00E-22	6.00E-10
	**OrthoMCL**	OG5_ 152674	OG5_ 127466	-	OG5_ 126554	OG5_ 126554	OG5_ 127252	n/a	OG5_ 129065	OG5_ 126690	OG5_ 126554	OG5_ 127112	n/a
	**ESG Confidence**	72.10%	100.00%	-	65.20%	65.10%	99.70%	n/a	46.80%	54.00%	53.80%	35.30%	n/a
	**Presence**	-	-/+	-	-/+	-/+	+	-	+	-/+	-/+	-/+	-
**EUSTIGMATO-PHYTE**													
Nannochloropsis oculata [d]	**Accession #**	none	none	none	none	none	none	none	none	none	none	none	none
	**E-value**	-	-	-	-	-	-	-	-	-	-	-	-
	**Reciprocal e-value**	-	-	-	-	-	-	-	-	-	-	-	-
	**OrthoMCL**	-	-	-	-	-	-	-	-	-	-	-	-
	**ESG Confidence**	-	-	-	-	-	-	-	-	-	-	-	-
	**Presence**	-	-	-	-	-	-	-	-	-	-	-	-
**CHRYSOPHYTE**													
Ochromonas danica [d]	**Accession #**	none	none	none	none	none	none	none	none	none	none	none	none
	**E-value**	-	-	-	-	-	-	-	-	-	-	-	-
	**Reciprocal e-value**	-	-	-	-	-	-	-	-	-	-	-	-
	**OrthoMCL**	-	-	-	-	-	-	-	-	-	-	-	-
	**ESG Confidence**	-	-	-	-	-	-	-	-	-	-	-	-
	**Presence**	-	-	-	-	-	-	-	-	-	-	-	-

**DINO-FLAGELLATES**														
Symbiodinium minutum [e]	**Accession #**	none	none	none	none	none	none	none	none	none	none	none	none
	**E-value**	-	-	-	-	-	-	-	-	-	-	-	-
	**Reciprocal e-value**	-	-	-	-	-	-	-	-	-	-	-	-
	**OrthoMCL**	-	-	-	-	-	-	-	-	-	-	-	-
	**ESG Confidence**	-	-	-	-	-	-	-	-	-	-	-	-
	**Presence**	-	-	-	-	-	-	-	-	-	-	-	-
**CRYPTO-PHYTE*S***													
Guillardia theta [a]	**Accession #**	XP_ 005831091	XP_ 005837094	XP_ 005825847	XP_ 005822842	XP_ 005822842	XP_ 005827081	XP_ 005827896	XP_ 00582109	none	XP_ 005822842	XP_ 005840936	XP_ 005837230
	**E-value**	3.00E-21	1.00E-15	4.00E-42	2.00E-21	8.00E-23	0.00E+00	7.00E-21	6.00E-37	-	2.00E-19	2.00E-25	3.00E-08
	**Reciprocal e-value**	2.00E-27	1.00E-15	4.00E-59	2.00E-50	2.00E-50	0.00E+00	0.00E+00	1.00E-37	-	2.00E-50	1.00E-24	2.00E-92
	**OrthoMCL**	OG5_ 129359	OG5_ 132214	OG5_ 146412	OG5_ 126554	OG5_ 126554	OG5_ 127252	OG5_ 126554	OG5_ 127644	-	OG5_ 126554	OG5_ 127112	n/a
	**ESG Confidence**	0.00%	100.00%	100.00%	62.40%	62.40%	99.80%	40.00%	0.00%	-	54.40%	36.10%	n/a
	**Presence**	+	-/+	-/+	-/+	-/+	+	-	-	-	-/+	-/+	-
Hemiselmis andersenii [d]	**Accession #**	none	none	none	none	none	none	none	none	none	none	none	none
	**E-value**	-	-	-	-	-	-	-	-	-	-	-	-
	**Reciprocal e-value**	-	-	-	-	-	-	-	-	-	-	-	-
	**OrthoMCL**	-	-	-	-								
	**ESG Confidence**	-	-	-	-	-	-	-	-	-	-	-	-
	**Presence**	-	-	-	-	-	-	-	-	-	-	-	-
**HAPTOPHYTE**													
Emiliania huxleyi CCMP1516 [d]	**Accession #**	XP_ 005764353	XP_ 005770598	XP_ 005759060	XP_ 005782001	XP_ 005793926	XP_ 005757560	XP_ 005793926	XP_ 00577640	XP_ 005758786	XP_ 005793926	XP_ 005780581	XP_ 005785520
	**E-value**	1.00E-37	2.00E-17	1.00E-48	3.00E-10	2.00E-11	7.00E-87	7.00E-30	7.00E-39	4.00E-91	2.00E-25	2.00E-64	2.00E-39
	**Reciprocal e-value**	1.00E-38	3.00E-26	8.00E-66	3.00E-24	4.00E-36	5.00E-137	5.00E-35	1.00E-39	5.00E-132	4.00E-36	6.00E-69	2.00E-41
	**OrthoMCL**	OG5_ 129359	OG5_ 132924	OG5_ 146412	OG5_ 127298	OG5_ 141359	OG5_ 127252	OG5_141359	OG5_ 129065	OG5_ 126690	OG5_ 141359	OG5_ 128985	OG5_ 131865
	**ESG Confidence**	58.50%	95.50%	50.30%	60.60%	73.90%	100.00%	72.40%	47.10%	62.60%	72.40%	67.40%	31.30%
	**Presence**	+/-	-/+	-/+	-/+	-/+	+	-	+	-/+	-/+	+	+

**ROSEOBACTER**													
Ruegeria sp. R11 [d]	**Accession #**	none	WP_ 008560074	none	none	none	WP_ 008561606	none	WP_ 00855800	none	none	WP_ 008563355	none
	**E-value**	-	5.00E-13	-	-	-	1E-95	-	3E-109	-	-	4.00E-27	-
	**Reciprocal e-value**	-	7.00E-19	-	-	-	2E-95	-	7E-109	-	-	7.00E-27	-
	**OrthoMCL**	-	OG5_ 127466	-	-	-	OG5_ 127252	-	OG5_ 129065	-	-	OG5_ 135328	-
	**ESG Confidence**	-	92.00%	-	-	-	100.00%	-	47.70%	-	-	99.50%	-
	**Presence**	-	-/+	-	-	-	+	-	+	-	-	-/+	-
Dinoroseobacter shibae DFL 12 [d]	**Accession #**	WP_ 012187434	WP_ 012177719	none	none	none	WP_ 012179620	WP_ 012179004	none	WP_ 012178326	WP_ 012179004	WP_ 012178466	none
	**E-value**	4E-23	1.00E-14	-	-	-	2E-104	4.00E-21	-	1E-54	4.00E-12	1.00E-16	-
	**Reciprocal e-value**	8E-27	2.00E-20	-	-	-	1E-103	1.00E-37	-	2E-58	9.00E-40	4.00E-16	-
	**OrthoMCL**	OG5_ 129359	OG5_ 127466	-	-	-	OG5_ 127252	OG5_ 126554	-	OG5_ 126690	OG5_ 126554	OG5_ 140291	-
	**ESG Confidence**	72.30%	100.00%	-	-	-	100.00%	52.40%	-	52.00%	52.40%	0.00%	-
	**Presence**	+/-	-/+	-	-	-	+	-/+	-	-	-/+	-	-
Jannaschia sp. CCS1 [d]	**Accession #**	WP_ 011456827	WP_ 011453552	none	none	none	WP_ 011454061	WP_ 011455517	WP_ 01145660	WP_ 011455066	none	WP_ 011457232	WP_ 011456849
	**E-value**	8E-38	9.00E-25	-	-	-	8E-100	7.00E-12	4E-95	4E-56	-	1.00E-27	2.00E-46
	**Reciprocal e-value**	2E-37	2.00E-27	-	-	-	2E-96	7.00E-40	5E-100	2E-64	-	2.00E-27	1.00E-45
	**OrthoMCL**	OG5_ 129359	OG5_ 132924	-	-	-	OG5_ 127252	OG5_126554	OG5_ 129065	OG5_ 126690	-	OG5_ 126697	OG5_ 131865
	**ESG Confidence**	39.70%	94.00%	-	-	-	100.00%	59.20%	46.90%	55_0%	-	37.30%	36.50%
	**Presence**	+/-	-/+	-	-	-	+	-/+	+	-	-	-/+	+
Phaeobacter gallaeciensis 2.10 [d]	**Accession #**	WP_ 014889329	WP_ 014881395	none	AFO87348	AFO87348	WP_ 014875707	AFO87348	WP_ 01487322	WP_ 014873899	none	WP_ 014889373	none
	**E-value**	8E-16	5.00E-19	-	3E-12	3.00E-12	2E-94	5.00E-15	2E-111	1E-60	-	3.00E-16	-
	**Reciprocal e-value**	8E-17	9.00E-19	-	5E-43	5.00E-43	4E-99	5.00E-43	4E-111	4E-64	-	1.00E-17	-
	**OrthoMCL**	OG5_ 126653	OG5_ 132924	-	OG5_ 126554	OG5_ 126554	OG5_ 127252	OG5_ 126554	OG5_ 129065	OG5_ 126690	-	OG5_ 126697	-
	**ESG Confidence**	63.30%	97.50%	-	47.30%	47.30%	100.00%	39.90%	47.10%	53.00%	-	47.00%	-
	**Presence**	-	-/+	-	-/+	-/+	+	-/+	+	-	-	-/+	-

Phaeobacter gallaeciensis DSM17395 (BS107) [d]	**Accession #**	WP_ 014881761	WP_ 014880496	none	AFO91064	AFO91064	WP_ 014880995	AFO91064	WP_ 01488122	WP_ 014879386	none	WP_ 014881859	none
	**E-value**	2E-15	3.00E-13	-	3E-12	3.00E-12	2E-94	2.00E-14	2E-110	2E-57	-	5.00E-16	-
	**Reciprocal e-value**	2E-16	5.00E-19	-	3E-40	3.00E-40	8E-99	1.00E-43	4E-110	2E-61	-	2.00E-17	-
	**OrthoMCL**	OG5_ 126653	OG5_ 127466	-	OG5_ 126554	OG5_ 126554	OG5_ 127252	OG5_ 126554	OG5_ 129065	OG5_ 126690	-	OG5_ 126697	-
	**ESG Confidence**	71.30%	94.00%	-	47.80%	47.80%	100.00%	37.90%	40.70%	64.00%	-	38.60%	-
	**Presence**	-	-/+	-	-/+	-/+	+	-/+	+	-	-	-/+	-
Roseobacter denitrificans OCh 114 [d]	**Accession #**	WP_ 011569789	WP_ 011566397	none	none	none	WP_ 011570123	WP_ 011567654	none	WP_ 011568514	WP0115676 54	WP_ 011566437	none
	**E-value**	2E-23	5.00E-15	-	-	-	1E-113	1.00E-14	-	1E-62	1.00E-15	4.00E-13	-
	**Reciprocal e-value**	2E-28	1.00E-19	-	-	-	6E-114	9.00E-48	-	2E-62	9.00E-48	8.00E-13	-
	**OrthoMCL**	OG5_ 129359	OG5_ 132924	-	-	-	OG5_ 127252	OG5_126554	-	OG5_ 126690	OG5_ 126554	OG5_ 135328	-
	**ESG Confidence**	48.30%	98.50%	-	-	-	99.80%	43.60%	-	53.00%	43.60%	100.00%	-
	**Presence**	+/-	-/+	-	-	-	+	-/+	-	-	-/+	-/+	-
Ruegeria pomeroyi DSS-3 [d]	**Accession #**	WP_ 011046547	WP_ 011047226	none	WP_ 011047630	WP_ 011047630	WP_ 011046404	WP_ 011047630	WP_ 01104935	none	WP_ 011047630	WP_ 030003254	WP_ 011241897
	**E-value**	4E-26	5.00E-20	-	1E-10	1.00E-10	1E-88	2.00E-16	3E-109	-	1.00E-12	6.00E-15	3.00E-43
	**Reciprocal e-value**	1E-25	7.00E-24	-	5E-46	5.00E-46	6E-95	5.00E-46	6E-109	-	5.00E-46	1.00E-14	7.00E-43
	**OrthoMCL**	OG5_ 126653	OG5_ 175845	-	OG5_ 126554	OG5_ 126554	OG5_ 127252	OG5_126554	OG5_ 129065	-	OG5_ 126554	OG5_ 135774	OG5_ 131865
	**ESG Confidence**	72.70%	96.50%	-	48.80%	48.80%	100.00%	39.90%	41.30%	-	39.90%	84.20%	35.90%
	**Presence**	-	-/+	-	-/+	-/+	+	-/+	+	-	-/+	-/+	+
Ruegeria sp. TM1040 [d]	**Accession #**	none	WP_ 011537675	none	none	none	WP_ 011540019	WP_ 011538791	WP_ 01153701	WP_ 011536924	WP_ 011538791	WP_ 011537211	none
	**E-value**	-	4.00E-22	-	-	-	4E-98	1.00E-18	1E-112	8E-57	1.00E-11	3.00E-17	-
	**Reciprocal e-value**	-	3.00E-25	-	-	-	4E-95	2.00E-43	3E-112	2E-58	2.00E-43	7.00E-17	-
	**OrthoMCL**	-	OG5_ 132924	-	-	-	OG5_ 127252	OG5_126554	OG5_ 129065	OG5_ 126690	OG5_ 126554	OG5_ 126697	-
	**ESG Confidence**	-	97.50%	-	-	-	98.20%	56.60%	47.10%	65.60%	56.60%	47.20%	-
	**Presence**	-	-/+	-	-	-	+	-/+	+	-	-/+	-/+	-
