# Supplementary material for: Indole-3-Acetic Acid Is Produced by Emiliania huxleyi Coccolith-Bearing Cells and Triggers a Physiological Response in Bald Cells
Source: Front Microbiol. 2016 Jun 8;7:828. doi: 10.3389/fmicb.2016.00828 (PMC4896954; doi:10.3389/fmicb.2016.00828)
Supplement: Supplementary file 3 [file Image1.pdf]

Supplementary Figure 1

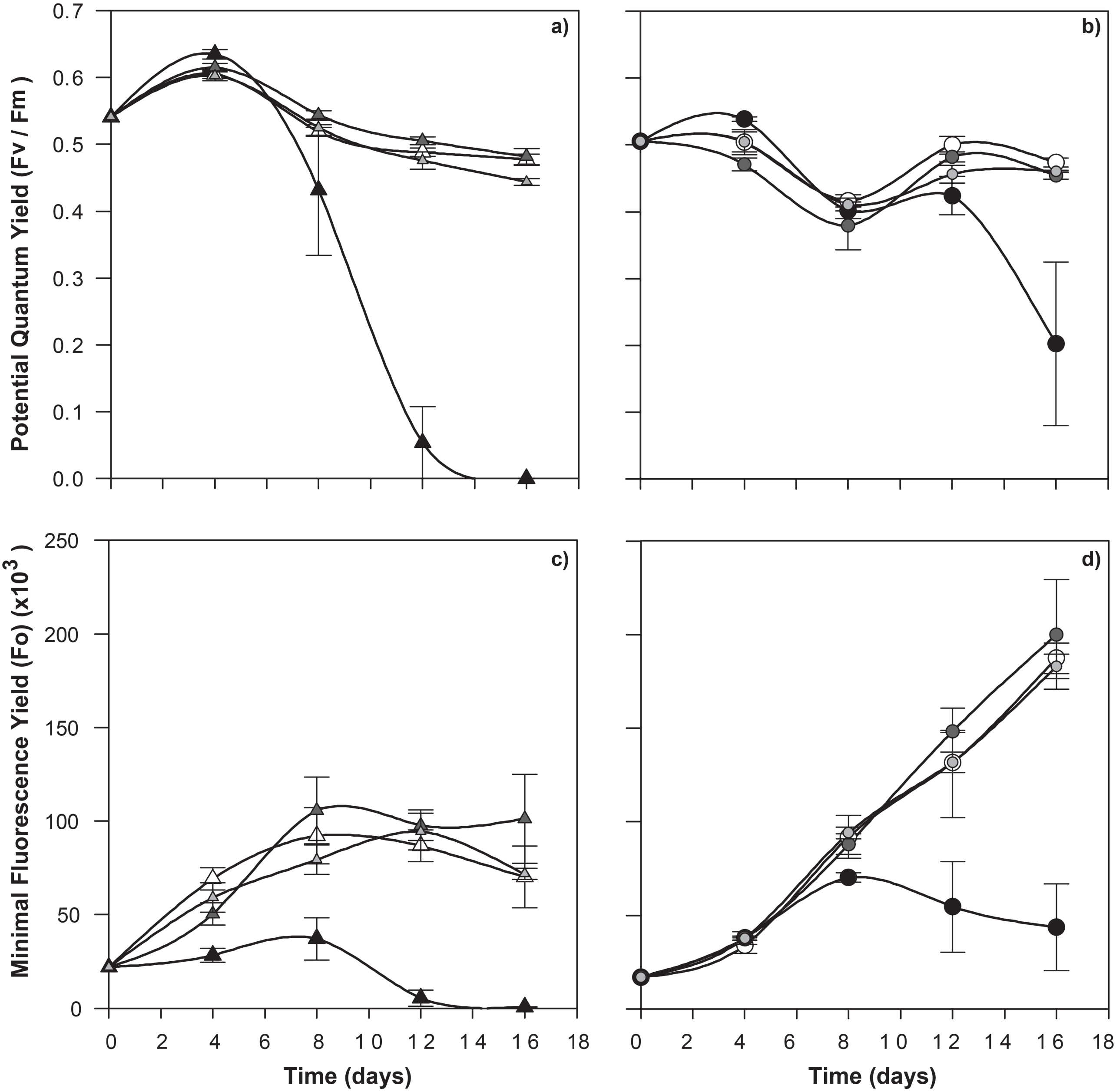

Supplementary Figure 1: Effect of exogenous L-tryptophan on axenic bald CCMP2090 and coccolith bearing CCMP3266 *E. huxleyi*. The algae were co-cultured with concentrations of 10^{-3} to 10^{-5} M of L-tryptophan (white for control samples with no additional tryptophan, black for 10^{-3} M, dark grey for 10^{-4} M and light grey for 10^{-5} M additional tryptophan). Triangles represent CCMP3266 while circles represent CCMP2090. The potential quantum yield of CCMP3266 (a) and CCMP2090 (b) with various concentrations of tryptophan is shown, while the minimal fluorescence for CCMP3266 (c) and CCMP2090 (d). Error bars represent ±1 SE.
